# NIR-II light-driven *in situ* nanovaccine for cancer immunotherapy via lymph node migration-mediated accumulation

**DOI:** 10.7150/thno.114347

**Published:** 2025-07-02

**Authors:** Yucheng Huang, Miao Su, Weihuan Lin, Peirong Zhang, Hengliang Hou, Fanjun Zeng, Luyu Huang, Qiaxuan Li, Jialong Deng, Shengbo Liu, Hongrui Qiu, Xiaoqing Yuan, Li Peng, Bin Xu, Haiyu Zhou

**Affiliations:** 1 School of Medicine, South China University of Technology, Guangzhou, 510006, P. R. China.; 2 Department of Thoracic Surgery, Guangdong Provincial People's Hospital (Guangdong Academy of Medical Sciences), Southern Medical University, Guangzhou, 510080, P. R. China.; 3 School of Biomedical Sciences and Engineering, South China University of Technology, Guangzhou International Campus, Guangzhou, 511442, P. R. China.; 4 Department of General Practice, Guangdong Provincial Geriatrics Institute, Guangdong Provincial People's Hospital (Guangdong Academy of Medical Sciences), Southern Medical University, Guangzhou, 510080, P. R. China.; 5 Department of Thoracic Surgery, Maoming People's Hospital, Maoming, 525000, P. R. China.; 6 Department of Surgery, Competence Center of Thoracic Surgery, Charité Universitätsmedizin Berlin, Berlin, 10117, Germany.; 7 Medical Research Center, Sun Yat-Sen Memorial Hospital, Sun Yat-Sen University, Guangzhou, 510120, P. R. China.

**Keywords:** nanovaccines, lymph node migration, viscous nanoparticles, immunotherapy

## Abstract

**Background:** Tumor vaccines that combine tumor antigens with immune adjuvants offer a promising strategy for cancer immunotherapy. A particularly effective approach involves the *in situ* generation of tumor antigens within the tumor microenvironment and their subsequent delivery to the lymph nodes alongside immune adjuvants. However, Co-delivery of antigen and adjuvant on a single nano-platform remains facing some key problems, such as a single nanomedicine cannot simultaneously achieve antigen production, capture, and delivery to lymph nodes, as well as leakage of adjuvant due to destruction of nano-drug by exogenous stimuli and failure to capture antigen effectively.

**Methods:** To address this, this study develops two synergistic nanodrugs: AuP and VNP_R848_. Under 1064 nm laser irradiation, AuP generates many tumor antigens, whereas VNP_R848_, distinguished by its small size and high viscosity, captures these autologous antigens and encapsulates the immune adjuvant R848.

**Results:** This combination creates an *in situ* cancer nanovaccine capable of lymph node migration. Nanovaccines enhance dendritic cell uptake and maturation, promoting antigen processing and presentation to T cells, which triggers a robust antitumor immune response. Remarkably, the nanovaccine we developed demonstrated superior therapeutic efficacy in distal tumors, postoperative recurrence and pulmonary metastatic tumor models, while also inducing long-term immune memory.

**Conclusion:** This study presents a straightforward and effective strategy for developing in situ nanovaccines for cancer immunotherapy, with wide-ranging clinical application prospects.

## Introduction

Inducing or enhancing T cell-mediated antitumor immune responses is widely regarded as one of the most promising strategies for eliminating tumor cells and preventing tumor recurrence and metastasis. This approach forms the basis for the development of therapeutic cancer vaccines 1-3. The essential principles for successful therapeutic cancer vaccines include delivering large quantities of high-quality personalized antigens to dendritic cells (DCs), optimizing DC activation, inducing robust and sustained cytotoxic T lymphocyte responses, promoting tumor microenvironment infiltration, and maintaining the persistence of these responses 4. These goals can be achieved through various methods, including reversing tumor-induced immune suppression using immune checkpoint inhibitors, activating DCs and effector T cells by co-administering tumor-associated antigens with adjuvants, and vaccinating autologous DCs loaded with specific tumor antigens. These approaches can broadly enhance the local tumor immune microenvironment, ultimately promoting tumor cell death 5,6. The DC-based vaccine SiPuleucel-T was clinically approved for treating advanced prostate cancer over a decade ago 7. However, it was later found that the survival advantage it provides to patients was minimal 8, leading to widespread disappointment in cancer vaccine development. In addition, a DCVax-L vaccine targeting brain cancer with patient immune cells failed to meet the overall survival endpoint and reported significant adverse effects in phase III clinical trials 9. A major reason for cancer vaccine failure is the selection of vaccine platforms or antigens with low immunogenicity 10. In tumors devoid of tumor-infiltrating T cells, commonly called “cold tumors”, therapeutically inducing effective antitumor immune responses remains particularly challenging 11. Therefore, the novel strategy utilizing light in the second near-infrared (NIR-II) window offers the potential for enhanced observation precision and superior tissue penetration depth in treating solid tumors 12. By utilizing NIR-II light-driven delivery of enhanced therapeutic energy selectively to deeper tumor tissues, this process transforms immunologically inert “cold tumors” into immunologically active “hot tumors”, thereby triggering the generation of abundant tumor antigens 13.

Most traditional cancer vaccines focus on specific antigens 14. However, the efficacy of these vaccines is significantly constrained by challenges such as the difficulty in identifying novel tumor antigens, inter- and intratumoral heterogeneity, inaccurate antigen identification, and antigen mutations 15-20. *In situ* cancer vaccines that convert autologous tumor tissue into personalized antigens have emerged as a promising approach for cancer vaccine development in recent years 21,22. Beyond antigens, cancer vaccines require adjuvants that can stimulate antigen-presenting cells (APCs) 23. An ideal tumor vaccine must achieve the simultaneous delivery of tumor antigens and adjuvants to the lymph nodes, along with internalization by APCs, to trigger a cascade of systemic immune responses 24.

Despite advances in cancer vaccine research, certain limitations remain, and current therapeutic outcomes fall short of meeting expectations. Consequently, nanotechnology-based nanovaccine platforms for personalized antigen generation and adjuvant delivery have garnered significant attention in recent years. Wang *et al.* utilized a hydrogel liposome system to efficiently encapsulate the Toll-like receptor (TLR) agonist imiquimod, enabling *in situ* vaccine functionality 25. Du *et al.* reported a nanodrug encapsulating the STING agonist MnO_2_, in which Mn^2+^ and antigens were formulated into subunits to induce *in situ* cancer vaccine effects 26. Most reported *in situ* vaccines are single nanoplatforms 27-30. However, single nanoplatforms face critical issues in the co-delivery of antigens and adjuvants, the process for individual nanoparticles to first enter tumor cells, induce antigen production, capture the antigens, and subsequently deliver them to lymph nodes is extremely complex and challenging to achieve. This is due to nanoparticles within tumor cells are susceptible to lysosomal/endosomal degradation 31, leading to premature leakage of adjuvants and failure to capture antigens, making the lymph node delivery process even more difficult to accomplish. Additionally, Immunotherapy induced by single nanoplatforms is prone to being hindered by low antigen presentation levels and a highly immunosuppressive tumor microenvironment 32. Due to insufficient antigen self-presentation by tumor cells and ineffective antigen cross-presentation by DCs, tumor cells evade immune recognition, thereby failing to effectively activate immune responses. Therefore, addressing how personalized antigen- and adjuvant-carrying nanovaccines can efficiently capture antigens, coordinate co-delivery with adjuvants to tumor-draining lymph nodes, and achieve internalization by APCs remains challenging.

**Figure 1:**
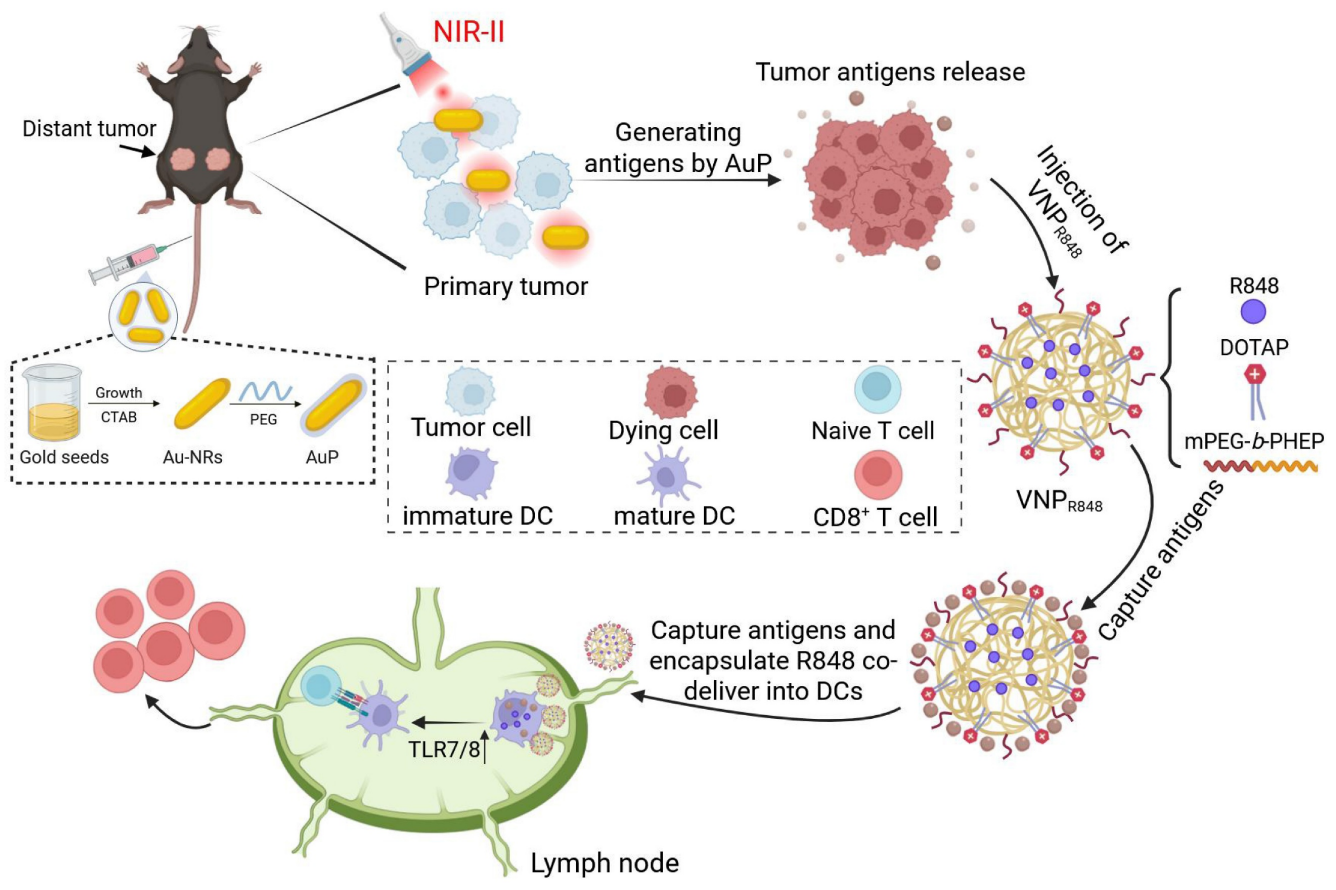
NIR-II-driven cancer nanovaccine for tumor immunotherapy. AuP produces a large amount of tumor antigens under 1064 nm laser irradiation. Subsequently, VNP_R848_ captures the generated antigens in the tumor tissue and co-delivers them to lymph nodes with the immune adjuvant R848, which is internalized by DCs. R848 then activates TLR 7/8, promoting the maturation of DC cells, activating CD8^+^ T cells, and inducing a robust antitumor immune response. Figure 1 was created with BioRender.com.

To address these challenges, we propose a straightforward, personalized, *in situ* tumor vaccine strategy. This approach leverages nanotechnology to eliminate *in situ* tumors, release antigens, and simultaneously transport antigens and immune adjuvants to the draining lymph nodes. This process initiates antigen presentation by DCs and subsequent antitumor immune responses (Figure 1). As the first step in *in situ* tumor antigen generation, we synthesized PEG-coated gold nanorods (AuPs) with high absorption in the Near-infrared II (NIR-II) window. Under 1064 nm laser irradiation, AuP exhibited excellent tissue penetration and a robust photothermal effect, effectively killing tumor cells and releasing abundant tumor antigens. To address the limitations of single nanoplatforms, particularly the challenging process spanning from tumor cell entry to lymph node delivery, we employed a secondary polymeric nanoparticle with an ultrasmall particle size (~28 nm). This nanoparticle efficiently delivered the immune adjuvant resiquimod (R848) to APCs in lymph nodes, ensuring effective antigen capture and adjuvant delivery. Owing to its viscous flow property and the presence of the cationic liposome DOTAP for capturing a substantial quantity of tumor-generated antigens, it was denoted as VNP_R848_. This *in situ* nanovaccine with lymph node-accumulated delivery capabilities triggered a robust antitumor immune response, completely inhibited or even eradicated certain distant tumors, and prevented tumor recurrence and metastasis. With a straightforward and scalable production process that enables potent therapeutic effects, this approach addresses the challenges of antigen capture and the co-delivery of antigens and immune adjuvants, providing a practical and effective technical strategy for developing personalized tumor vaccines.

## Results and Discussion

### Design and characterization of synergistic nanovaccines

**Figure 2:**
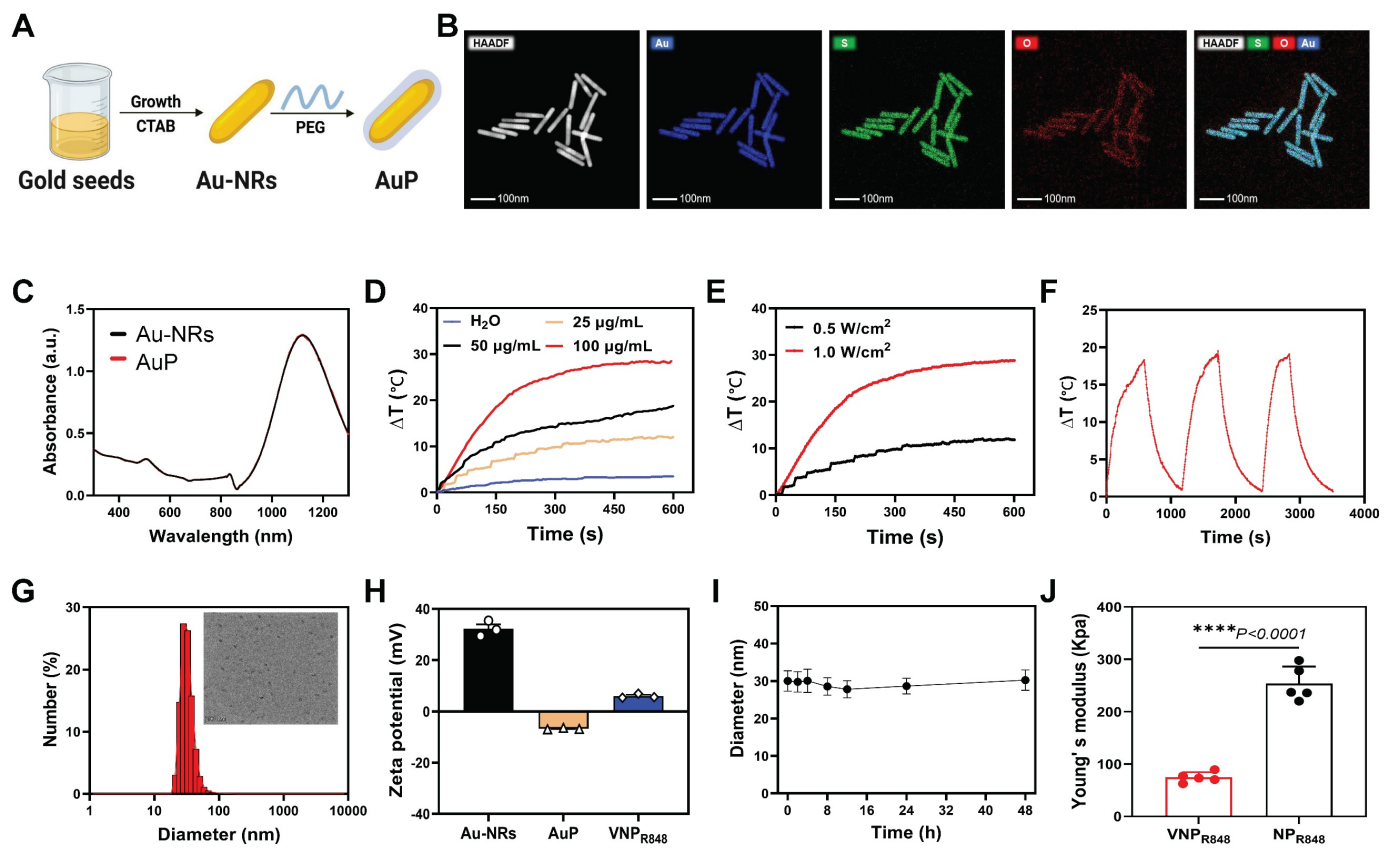
Preparation and characterization of AuP and VNP_R848_. (A) Schematic illustration of the preparation of Au-NRs and PEG modification to generate Au-PEG. (B) Scanning transmission electron microscopy and energy dispersive spectroscopy mapping of AuP. Scale bar: 100 nm. (C) Absorption spectrum of Au-NRs and AuP. (D) Variation in the AuP solution temperature under 1064 nm laser irradiation (0.5 W cm^-2^). (E) Variation in the temperature of the AuP solution (25 µg mL^-1^) under various 1064 nm laser intensities. (F) Photothermal conversion stability of the AuP solution under 1064 nm laser irradiation. (G) Size distribution and representative TEM images of VNP_R848_. Scale bar: 200 nm. (H) Zeta potentials of Au-NRs, AuP, and VNP_R848_ (n = 3). (I) Colloidal stability of VNP_R848_ in PBS (n = 3). (J) Young's modulus values of VNP_R848_ and NP_R848_ (n = 5). Data are shown as the mean ± standard deviation; the comparison of two groups was followed by Unpaired student's t-test (two-tailed). ****P < 0.0001. Image in A was created with BioRender.com.

Gold nanorods, with their high absorption capability in the NIR-II window, exhibit efficient photothermal performance 33-35. Therefore, they have been used as carriers for personalized antigen production. In this study, gold nanorods were prepared using the seed growth method 36, and a polyethylene glycol (PEG) coating was used to remove the toxic stabilizer cetyltrimethylammonium bromide (CTAB) from the surface of the gold nanorods to obtain PEGylated gold nanorods 37, named AuP (Figure 2A). Element co-positioning presented the AuP structure with a long aspect ratio of 10.0 (length, 100 ± 10.0 nm; width, 10 ± 0.5 nm) (Figure 2B); thus, AuP had a robust absorbance in the NIR-II spectrum (Figure 2C). The prepared AuP produced a manageable photothermal property under 1064 nm laser irradiation, with its concentration (Figure 2D) and the laser efficiency (Figure 2E) regulating its temperature. Additionally, the prepared AuPs exhibited excellent photothermal stability after three on/off laser cycles (Figure 2F).

VNP_R848_ preparation used amphiphilic poly-(ethylene glycol)-*block*-poly(2-hexoxy-2-oxo-1,3,2-dioxaphospholane) (mPEG-*b*-PHEP, Figure S1), which is known for its excellent viscosity 38-40, to encapsulate R848, hence the name VNP_R848_. Studies have shown that positively charged and large surface areas can efficiently capture proteins via electrostatic interactions 41,42. Therefore, the cationic liposome DOTAP (10% w/w) was added to enhance the ability of the nanoparticles to capture antigens. After optimization, the size of VNP_R848_ determined via dynamic light scattering (DLS) was 28 nm and Transmission electron microscopy (TEM) analysis exhibited the uniform distribution of particles and spherical morphology of VNP_R848_ (Figure 2G). Moreover, the zeta potentials of Au-NRs, AuP, and VNP_R848_ were +32.2 mV, -6.72 mV and +5.62 mV, respectively (Figure 2H). The charge changes proved that PEG was indeed coated onto the gold nanorods. More importantly, VNP_R848_ exhibited excellent colloidal stability for 48 h (Figure 2I).

The glass transition temperature (T_g_) of mPEG-PHEP was -54.32 °C, significantly lower than ambient conditions, indicating that it existed in a viscous flow state with fluidity at room temperature (Figure S2A). As a control, the commercially available mPEG-PLA with the same molecular weight exhibited a T_g_ of 30.28 °C (Figure S2B). In contrast, the rigid mPEG-PLA with a Tg of 30.28°C maintained a glassy state at body temperature and demonstrated rigidity under ambient conditions. The thermomechanical properties of the nanoparticles were influenced by the composition of their core nanostructures. Therefore, to demonstrate the viscosity and deformability of VNP_R848_, mPEG-PLA nanoparticles encapsulating R848 were first prepared using the same synthesis method as a comparative group, designated as NP_R848_. The preparation process was adjusted to endow it with similar particle size and Zeta potential to VNP_R848_ (Figure S3A-B). Evaluation of the Young's modulus of both particles via atomic force microscopy (AFM) revealed that VNP_R848_ exhibited a significantly lower Young's modulus compared to NP_R848_ (Figure 2J). This reduced Young's modulus conferred deformability to VNP_R848_, facilitating its transport to lymph nodes. Additionally, DOTAP-free VNP_R848_(-), as well as DOTAP-containing NP_R848_ and VNP_R848_, were respectively co-incubated with BSA protein to validate the protein-capturing capacity of the incorporated DOTAP. The results demonstrated that the addition of positively charged DOTAP nanoparticles significantly increased protein capture, while VNP_R848_ captured more protein owing to its excellent viscous flow properties (Figure S3C). Subsequently, the release kinetics of R848 from VNP_R848_ were quantitatively measured at pH levels of 5.5 and 7.4. The results demonstrated that nearly 80% of R848 was released from the nanoparticles under pH 5.5, with a significantly higher release rate compared to pH 7.4, indicating accelerated drug release kinetics of VNP_R848_ in acidic environments (Figure S4). This enhanced release facilitates R848 delivery within the endosomal/lysosomal compartments of DCs, thereby activating the TLR7/8 signaling pathway.

### Antigens capture and stimulate DC maturation by AuP/VNP_R848_

**Figure 3:**
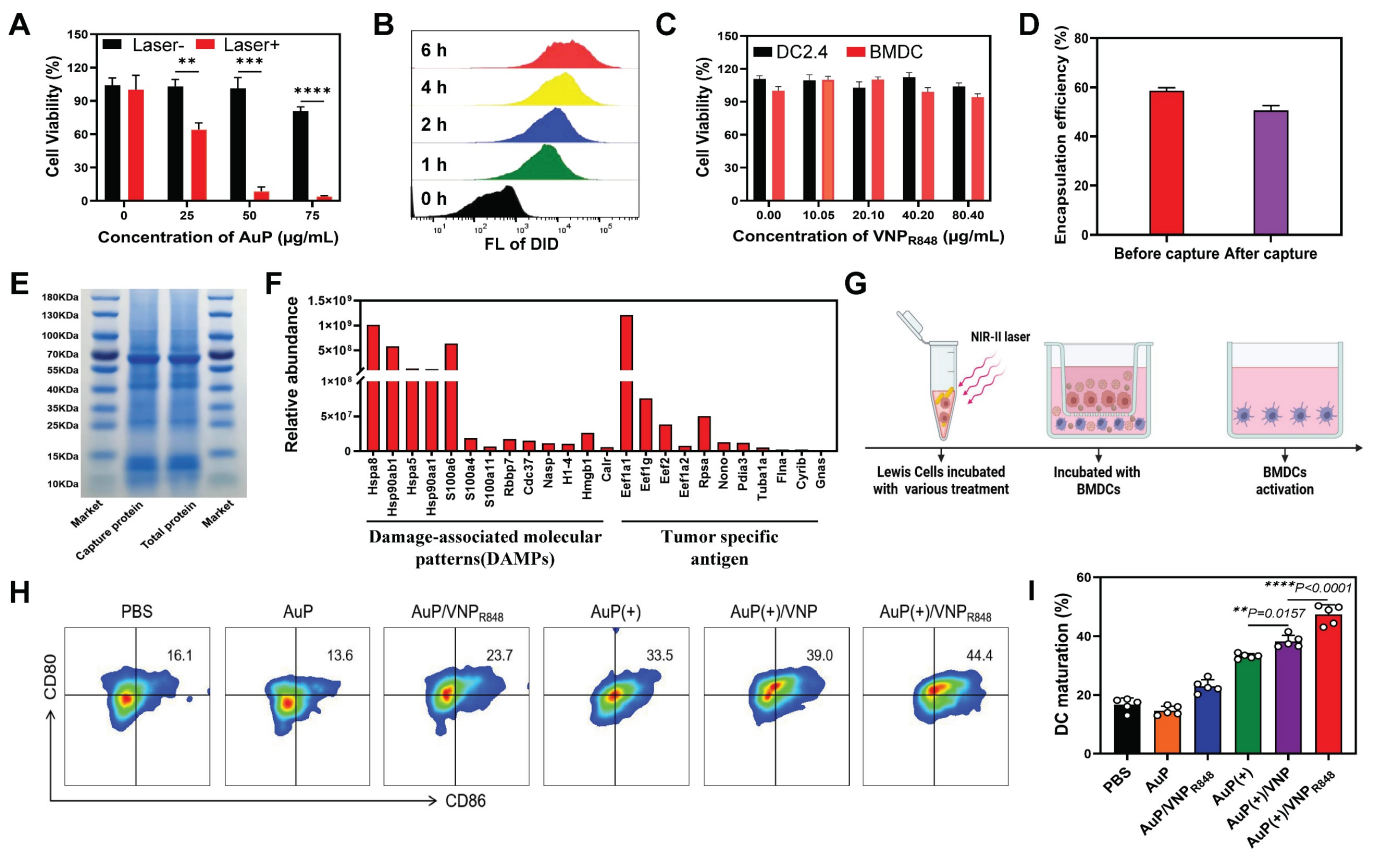
Antigens capture and stimulate DC maturation by AuP/VNP_R848_. (A) Results of the CCK8 cell viability assay. Different concentrations of AuP were incubated with LLC cells for 4 h and irradiated with a 1064 nm laser (0.5 W cm^-2^, 10 min) (n = 3). (B) Flow cytometry histogram of bone marrow-derived cells (BMDCs) treated with DiD-labeled VNP. (C) Cell viability of DC2.4 cells and BMDCs incubated with VNP_R848_ for 24 h as measured using a CCK8 assay. (D) Encapsulation rate of R848 before and after protein capture by VNP (n = 3). (E) SDS-PAGE analysis of the total proteins generated by AuP and the proteins captured by VNP_R848_ under 1064 nm laser irradiation. (F) Relative abundance of tumor antigens captured by VNP_R848_ as determined using liquid chromatography/tandem mass spectrometry. (G) Schematic illustration of dendritic cell (DC) maturation *in vitro* in a Transwell co-culture system. LLC cells subjected to different pretreatments were seeded in the upper chamber, and BMDCs extracted from mice were seeded in the lower chamber. (H) Representative flow cytometry plots and (I) proportions of mature DCs (CD11c^+^CD80^+^CD86^+^) induced by various treatments *in vitro* (n = 5). (+) represents 1064 nm laser irradiation (0.5 W cm^-2^, 10 min). Data are shown as the mean ± standard deviation. Statistical significance was analyzed using an unpaired Student's t-test (two-tailed) (A) or one-way ANOVA followed by Tukey's multiple comparisons test (I). *P < 0.05, **P < 0.01, ***P < 0.001, ****P < 0.0001. Image in G was created with BioRender.com.

In this study, we found that PEGylated gold nanorods were essentially non-toxic to cells, albeit at a concentration of 75 µg mL^-1^ (Figure S5). Moreover, they could directly induce tumor cell death under exposure to a 1064 nm laser (10 min, 0.5 W cm^-^²) (Figure 3A), potentially generating a substantial amount of antigens.

The ability of VNP_R848_ to enter DCs was also analyzed using flow cytometry. Using DiD to label VNP particles (VNP_DID_), the intracellular fluorescence signal increased significantly as the incubation time increased (Figure 3B and S6), indicating that bone marrow-derived cells (BMDCs) could effectively uptake VNP particles. Moreover, VNP_R848_ exhibited negligible effects on the viability of BMDCs and DC2.4 as determined using a CCK8 assay (Figure 3C). Next, we verified that AuP could generate antigens and be captured by VNP_R848_ while efficiently encapsulating the adjuvants. LLC cells were incubated with AuP and then exposured with a 1064 nm laser. Subsequently, VNP_R848_ was added to capture released *in situ* tumor antigens. To confirm that the nanoparticles effectively encapsulated R848 after capture, the content of R848 in VNP_R848_ before and after antigen capture was analyzed. The results indicated that following antigen capture, the VNP_R848_ maintained an encapsulation rate of 50.6%, similar to the pre-capture rate of 58.6% (Figure 3D). Next, the total protein generated and the protein captured by VNP_R848_ were assembled and analyzed via gel electrophoresis. The results showed that the AuP-mediated photothermal effect generated many proteins related to tumor antigens that were efficacious captured by VNP_R848_ (Figure 3E). After capturing proteins, the particle size of the VNP_R848_ increased vaguely from 28 nm to 35 nm (Figure S7A), and its zeta potential declined from +5.62mV to -1.15mV (Figure S7B). Quantitative analysis indicated that VNP_R848_ could effectually capture proteins released by tumor cells, with a capture amount of 83.2 μg protein per mg nanoparticle (Figure S7C). In addition, the results of mass spectrometry analysis demonstrated that VNP_R848_ effectively captured the tumor-specific antigens Eef1a1, Eef2, and Gnas, and damage-associated molecular patterns (DAMPs) Hsp90ab1, Hsp90aa1, Hspa8, and Hmgb1 (Figure 3F). These results suggest that VNP_R848_ can effectively encapsulate R848 after capturing large amounts of tumor antigens. Moreover, they indicate that plentiful tumor antigens can be produced under NIR-II light treatment and that the released tumor antigens can be captured by VNP_R848_ without affecting the drug loading and antigen capture functions of the nanoparticles, ultimately forming an *in situ* cancer vaccine with antigens and adjuvants.

Encouraged by these results, we further explored the ability of cancer nanovaccines to activate DCs. By performing a Transwell assay, AuP was added to LLC cells for incubation and subsequently treated with laser irradiation, followed by the addition of VNP_R848_, while BMDCs were added to the lower chamber and incubated for 24 h (Figure 3G). Flow cytometry analysis showed that the AuP/VNP_R848_ and AuP(+) treatment groups exhibited general immunogenicity, stimulating DC maturation by approximately 23.7% and 33.5%, respectively (Figure 3H-I). Notably, compared with the AuP(+) group, the AuP(+)/VNP group exhibited a superior DC maturity (39%), which may be due to the fact that VNP without immune adjuvants can capture antigens and deliver them to DCs to stimulate DC maturation. Most importantly, the DC maturity of the AuP(+)/VNP_R848_ group was the highest at 44.4%. This demonstrated that these nanovaccines could effectively stimulate and activate DCs.

### Lymph node accumulation and antigen presentation ability of *in situ* nanovaccines *in vivo*

**Figure 4:**
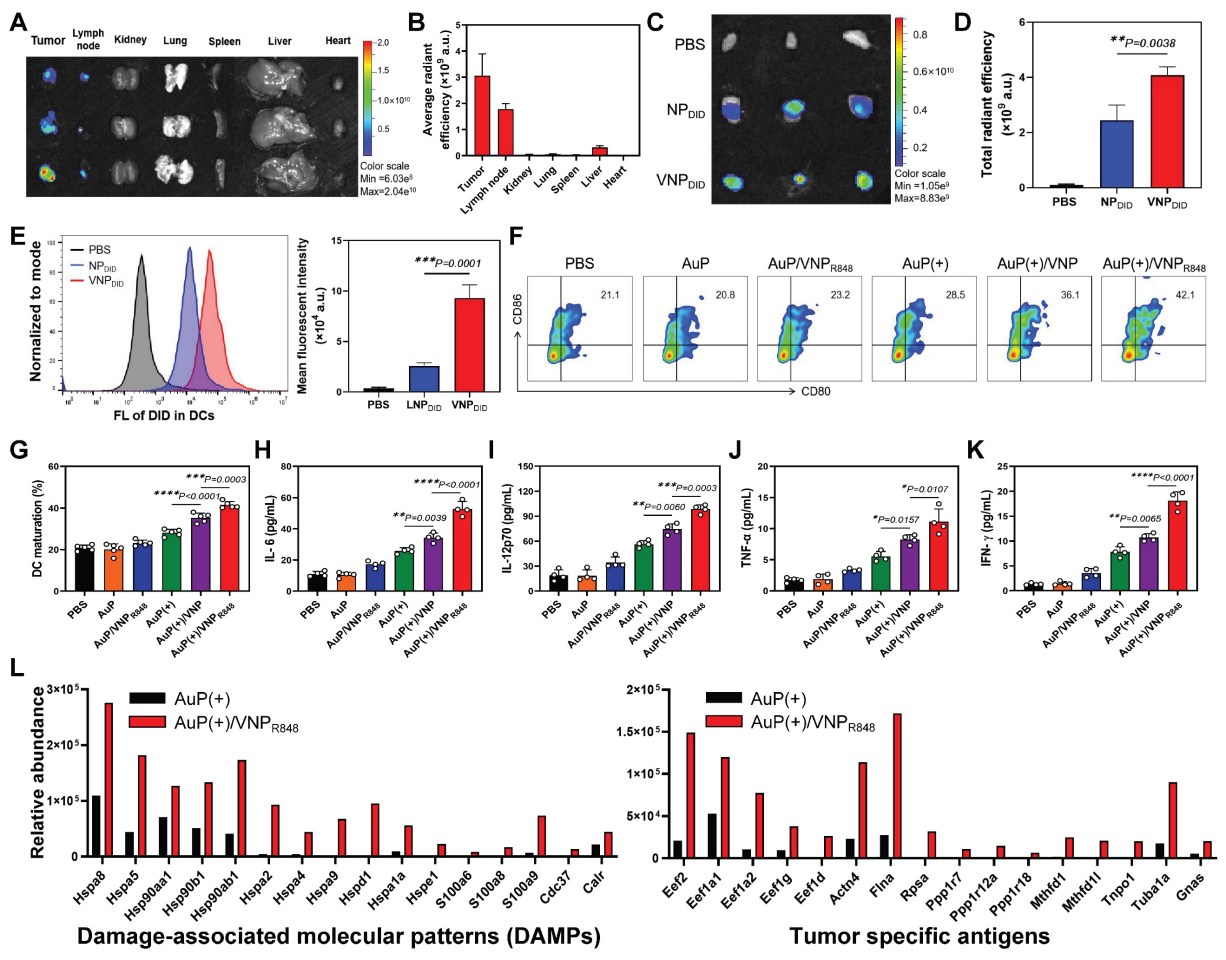
*In situ* nanovaccines for lymph node delivery and induced immune responses *in vivo*. (A) *Ex vivo* imaging of major organs and tumor tissues was performed 24 h after VNP_DID_ injection. (B) Quantification of DiD fluorescence signals collected in (A) (n = 3). (C) *In vivo* imaging system analysis of the accumulation of NP_DID_ and VNP_DID_ in tumor-draining lymph nodes. (D) Quantification of DiD fluorescence signals from the lymph nodes collected in (C) (n = 3). (E) Representative flow cytometry histogram and corresponding proportions of DiD-positive DCs in lymph nodes (n = 3). (F) Representative flow cytometry plots of DCs isolated from the tumor-draining lymph nodes of LLC tumor-bearing mice. (G) Quantification of the proportions of mature DCs in (E) (n = 5). (H-I) Serum cytokine concentrations in mice receiving various treatments as measured using ELISA (n = 5). (L) Identification of damage-associated molecular patterns and tumor antigens bound to MHC-I molecules within the lymph nodes using immunopeptidomics. (+) represents 1064 nm laser irradiation (0.5 W cm^-2^, 10 min). Data are shown as the mean ± standard deviation. Statistical significance was analyzed using an unpaired Student's t-test (two-tailed) (D) or one-way ANOVA followed by Tukey's multiple comparisons test (G-K). *P < 0.05, **P < 0.01, ***P < 0.001, ****P < 0.0001.

We evaluated the capability of the nanovaccine to migrate to the lymph nodes and be assimilated by DCs, a key prerequisite for initiating a CD8^+^ T cell-mediated immune response. Systemic distribution and lymph node accumulation were determined 24 h after intratumoral administration applying an *in vivo* imaging system (IVIS). The results indicated that VNP_DID_ predominantly accumulated in the tumors and lymph nodes 24 h after intratumoral administration (Figure 4A-B). To better investigate the migration of nanoparticles to lymph nodes and their internalization by APCs, DID-labeled NP_DID_ and VNP_DID_ were employed. Individual analysis of lymph nodes via IVIS revealed that the fluorescence signals were much stronger in the VNP_DID_ group compared to the NP_DID_ group (Figure 4C-D). VNP_DID_ demonstrated effective accumulation in lymph nodes benefiting from its optimized size and deformability, while NP_DID_, despite its comparable particle sizes to VNP_DID_, showed partial migration to lymph nodes. The uptake of nanoparticles by DCs in lymph nodes (CD45^+^CD11^+^) was further assessed via flow cytometry analysis. The results indicated that the uptake of VNP_DID_ by DCs was significantly higher than that of NP_DID_ (Figure 4E). *In vivo* biological distribution analysis also showed that approximately 10% of R848 cells migrated to the lymph nodes (Figure S8A). In addition, AuP was effectively enriched in the tumor tissues 24 h after intravenous administration (Figure S8B). To validate that AuP can provide guidance for cancer diagnosis and treatment, PAM imaging was conducted.

Photoacoustic microscopy (PAM) imaging, an emerging hybrid technology, combines the advantages of optical and ultrasonic imaging to provide high optical resolution and deep acoustic penetration 43. This technique relies on contrast agents with high molar absorption coefficients in the NIR-I/NIR-II regions to generate sufficient optical absorption, creating contrast with surrounding tissues. Notably, nearly all reported gold nanoparticles currently operate within the NIR-I window (650-900 nm) 44-47. Compared to NIR-I photoacoustic imaging, NIR-II photoacoustic imaging exhibits higher signal-to-noise ratios, deeper tissue penetration depths, and superior spatiotemporal resolution. PAM imaging was performed using two distinct wavelengths (532 nm and 1064 nm) to achieve high-sensitivity visualization of the microvascular system in tumor regions and precise tracking of nanotheranostic agents, respectively. At 532 nm, the dense vascular network within the tumor area enabled PAM imaging to clearly delineate the tumor boundaries from surrounding tissues. In contrast, the 1064 nm wavelength, owing to its lower pulse energy and weaker blood absorption, failed to exhibit distinct vascular features. Following intravenous injection, incremental accumulation of AuP in the tumor was observed, reaching a peak at 24 hours post-injection, 3D visualization diagrams and quantitative data confirmed the enhanced AuP signal in tumors (Figure S9), providing precise guidance for cancer diagnosis and treatment.

We subsequently examined the effects of the nanovaccine on DC maturation and immune cytokine secretion and verified that the nanovaccine could capture antigens *in vivo* and deliver them to the lymph nodes. LLC subcutaneous lung cancer mice were treated with PBS, AuP, AuP/VNP_R848_, AuP(+), AuP(+)/VNP, or AuP(+)/VNP_R848_. Tumor-draining lymph nodes were removed 3 days post-treatment, and DC maturity was investigated using multichannel flow cytometry (Figure S10). Compared with other groups, the percentage of DC maturity (CD80^+^CD86^+^) in lymph nodes of the AuP(+)/VNP_R848_ group (42.1%) was significantly increased (Figure 4F-G). Additionaly, enzyme-linked immunosorbent assays (ELISAs) were utilized to detect IL-6, IL-12p70, IFN-γ, and TNF-α at 72 h after treatment (Figure 4H-K). Compared to the PBS group, samples not illuminated by NIR-II light had little effect on the secretion of immune cytokines. AuP(+) slightly increased immune cytokine secretion levels, while AuP(+)/VNP_R848_ treatment significantly enhanced the secretion of these cytokines, resulting in the highest levels of immune cytokine secretion.

T cells discern antigens present in major histocompatibility complex (MHC) molecules, thereby initiating immune responses. Immunopeptidomics, which employs mass spectrometry (MS), allows the analysis of MHC-bound peptides in cells or tissues to provide valuable information for the development of cancer vaccines. Consequently, identifying tumor antigens presented by MHC molecules is crucial for designing effective and safe cancer vaccines that harness T cells to specifically target and eliminate tumor cells 48-50. We first measured the relative abundance of DAMPs and tumor antigens generated in tumor tissues. The results demonstrated that AuP under NIR-II irradiation significantly induced the production of plentiful DAMPs and tumor antigens in tumor tissues (Figure S11). To demonstrate that VNP_R848_ could capture antigens, transport them into lymph nodes, and be internalized by APCs, we administered AuP(+) or AuP(+)/VNP_R848_ to tumor-bearing mice. Following treatment, we isolated the lymph nodes after 24 h and employed an MS-based immunopeptidomics analysis to detect the presence of tumor antigens bound to MHC-I within the lymph nodes (Figure 4L). The results identified a large number of DAMPs and tumor-specific antigens presented by MHC-I molecules in the lymph nodes of the AuP(+)/VNP_R848_ group, whereas only a minimal amount was examined in the AuP(+) group. These results suggest that VNP_R848_ can transport antigens to lymph nodes, triggering a cascade of immune responses. Collectively, these results indicate that treatment with AuP(+)/VNP_R848_ nanovaccines with lymph node-accumulating capability efficiently captured antigens at tumor lesions and delivered them to the lymph nodes, significantly activated the innate immune system, enhanced the maturation of DCs *in vivo*, and stimulated the release of pro-inflammatory cytokines.

### *In vivo* antitumor efficacy and potential immune mechanisms of *in situ* nanovaccines

**Figure 5:**
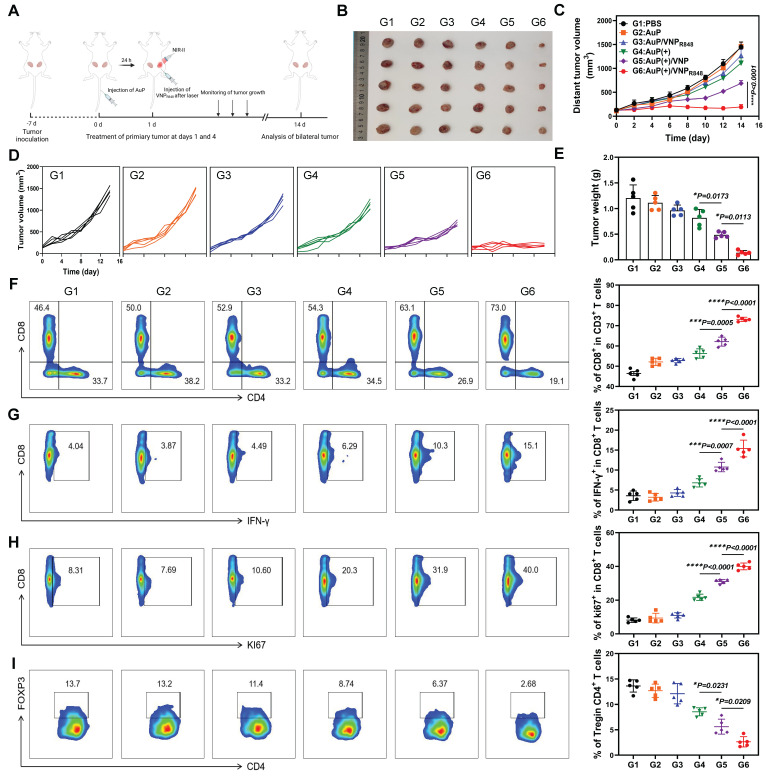
Antitumor effects of *in situ* nanovaccines and prospective immune mechanisms. (A) Experimental protocol in a bilateral LLC lung tumor model. (B) Photographs of *ex vivo* LLC tumors from mice that received various treatments. (C) Average and (D) individual distant tumor growth curves after various treatments (n = 5). (E) Weights of tumors removed from the mice at the end of treatment (n = 5). (F-H) Representative flow cytometry plots and proportions of CD8^+^ T cells (among CD45^+^CD3^+^ cells) (F), Ki67^+^CD8^+^ T cells (among CD8^+^ cells) (G), and IFN-γ^+^CD8^+^ T cell (among CD8^+^ cells) (H) in the tumor tissues (n = 5). (H) Representative flow cytometry plots and proportions of Treg cells (n = 5). Data are shown as the mean ± standard deviation. Statistical significance was analyzed using one-way ANOVA followed by Tukey's multiple comparisons test. *P < 0.05, **P < 0.01, ***P < 0.001, ****P < 0.0001. Image in A was created with BioRender.com.

Inspired by the capability of the nanovaccine to stimulate DC maturity and increase the release of pro-inflammatory cytokines, its *in vivo* therapeutic effects were further evaluated. LLC cells were inoculated subcutaneously on the right side of mice, and a second tumor was inoculated on the left side to simulate tumor metastasis. At 7 days post-inoculation, the mice were stochastically assigned to six groups: (G1) PBS, (G2) AuP, (G3) AuP/VNP_R848_, (G4) AuP(+), (G5) AuP(+)/VNP, and (G6) AuP(+)/VNP_R848_ (Figure 5A). AuP was intravenously (*i.v.*) injected before 24 h of laser irradiation, and then the primary tumors were intratumorally (*i.t.*) injected with VNP_R848_. Groups G4, G5, and G6 received a 1064 nm laser irradiation 24 h after an *i.v.* AuP injection. After NIR-II laser treatment, the photothermal effect *in vivo* was remarkable, and the temperature increased rapidly (Figure S12A-B). The groups exposed to NIR-II light prominently restrained the progression of primary tumors, whereas the AuP(+)/VNP_R848_ treatment group outright impeded the progression of primary tumors and even eradicated some primary tumors (Figure S13A-D). Notably, the progression of distant tumors in the *in situ* nanovaccine group treated with AuP(+)/VNP_R848_ was also completely inhibited, with minimal regrowth observed. (Figure 5B-E). As a control, the AuP(+)/VNP treatment group, without R848, also moderately inhibited the growth of distant tumors, demonstrating the prominent function of immune adjuvants in the antitumor immune response within the lymph nodes 51. Notably, the body weights of mice remained largely unchanged throughout the treatment period (Figure S14). Besides, hematoxylin and eosin (H&E) staining of the principal organs (Figure S15) and analysis of blood biochemical indices (Figure S16) revealed no abnormalities, indicating that the nanovaccine possesses excellent biological safety.

To further illumine the potential immune mechanisms of the nanovaccine, distant tumors were harvested for flow cytometry analysis after treatment, and the ratio of CD8^+^ T cells (CD45^+^CD3^+^CD8^+^) in the distal tumors was analyzed (Figure 5F and S17). The infiltration of CD8^+^ T cells in AuP(+)/VNP-treated mice was moderately increased, whereas the proportion of tumor-infiltrating CD8^+^ T cells in AuP(+)/VNP_R848_-treated mice increased dramatically to ~70% compared to that in the PBS group (~40%). Interestingly, the interferon-gamma (IFN-γ) release ability (CD8^+^IFN-γ^+^) (Figure 5G) and hyperplasia (Figure 5H) ratio of CD8^+^ T cells (CD8^+^KI67^+^) in distal tumors in the AuP(+)/VNP_R848_ treatment group also increased remarkably. Furthermore, the proportion of Tregs (CD4^+^Foxp3^+^) (Figure 5I) was notably reduced in the AuP(+)/VNP_R848_ treatment group, exhibiting an enhanced immunosuppressive tumor microenvironment.

**Figure 6:**
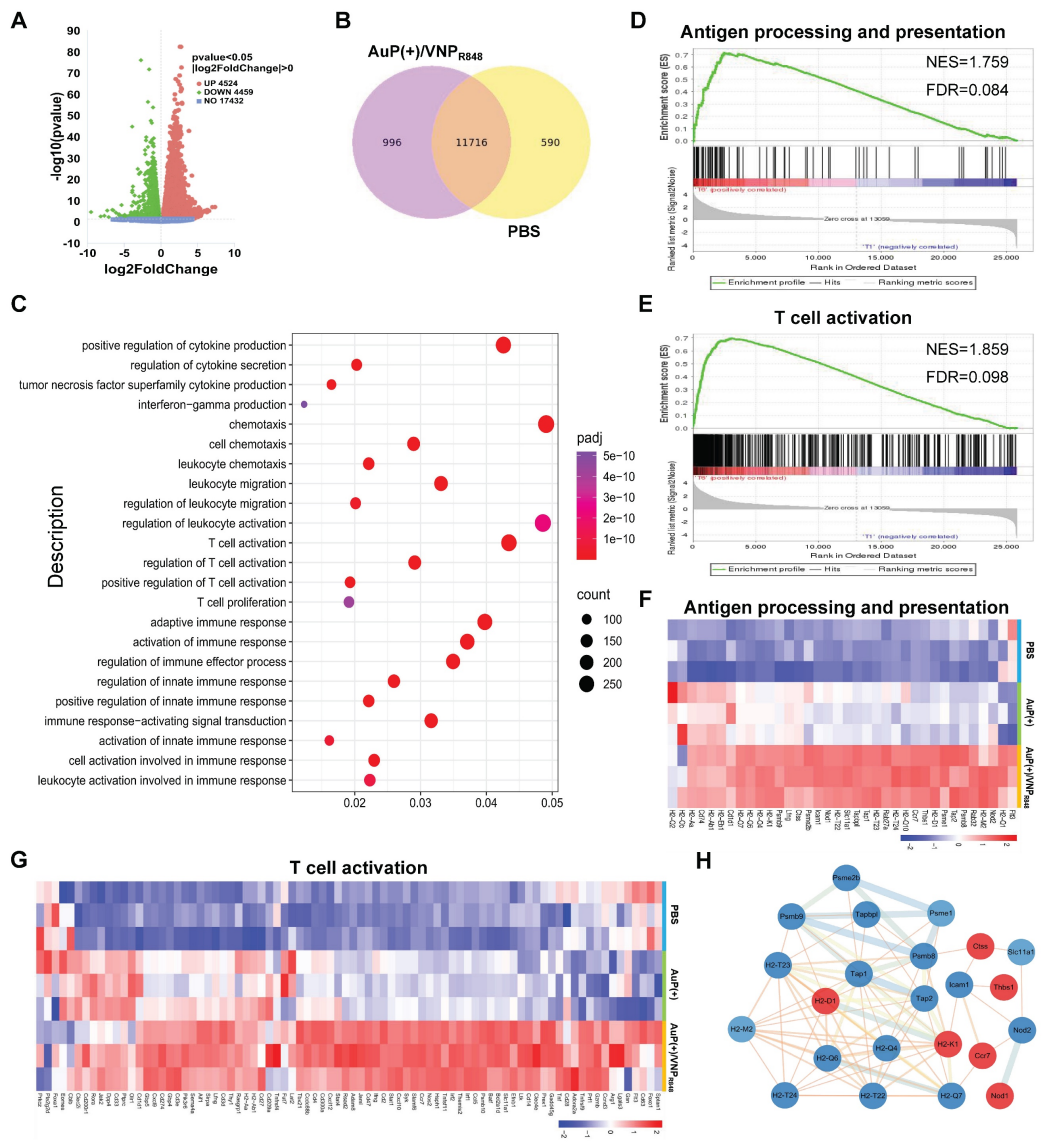
Transcriptomic sequencing analysis of distant tumors after *in situ* nanovaccine treatment. (A) Volcano plot showing the differentially expressed genes in the AuP(+)/VNP_R848_ group compared with the PBS group. (B) Venn diagram of genes co-expressed and exclusively expressed in the two groups. (C) Gene ontology analysis of the considerably upregulated and downregulated genes in the AuP(+)/VNP_R848_ group. (D, E) Gene set enrichment analysis for antigen processing and presentation (D) and T cell activation (E) signaling pathway after AuP(+)/VNP_R848_ treatment. (F, G) Heatmap of the differentially expressed genes related to antigen processing and presentation (F) and T cell activation (G). (H) Protein-protein interaction network associated with the antigen processing and presentation signaling pathways.

To sufficiently analyze the antitumor immune principle exerted by the cancer nanovaccines, distant tumor tissues were harvested for next-generation transcriptomic analyses. Compared with the PBS group, 8983 genes in the AuP(+)/VNP_R848_ group exhibited significant expression differences (Figure 6A), including 4524 upregulated genes and 4459 downregulated genes. The Venn diagram (Figure 6B) revealed that 996 genes were specifically differentially expressed in the AuP(+)/VNP_R848_ group. Gene ontology (GO) enrichment analysis was then implemented to elucidate the role and regulation mechanisms of the altered genes *in vivo* (Figure 6C). The results indicated that the AuP(+)/VNP_R848_ nanovaccine group was significantly enriched in pathways related to positive regulation of cytokine production, T cell activation, innate immunity, and adaptive immune response. Furthermore, Kyoto Encyclopedia of Genes and Genomes (KEGG) pathway investigation (Figure S18) revealed that the AuP(+)/VNP_R848_ nanovaccine group significantly activated the Toll-like receptor and NF-κB signaling pathways, with the upregulated genes being involved in the T cell receptor signaling pathway, antigen processing and cross-presentation, inflammatory response, cytokine-cytokine receptor interaction, and other pathways. The distribution trendency of immune-related pathway gene sets in the PBS and AuP(+)/VNP_R848_ groups was further evaluated using gene set enrichment analysis. The results exhibited that after treatment, the nanovaccine group showed an increasing trend in antigen processing and presentation, T cell activation, and other immune response-relevant pathway genes (Figure 6D-E and S19), revealing that these immune-related signaling pathways were triggered during nanovaccine treatment.

Heatmap analysis demonstrated that the genes related to antigen processing and presentation (Figure 6F) and T cell activation (Figure 6G) were prominently upregulated after AuP(+)/VNP_R848_ treatment. Moreover, protein-protein interaction network analysis was conducted on a set of genes related to antigen processing and presentation (Figure 6H), revealing that these genes were closely related and had complex interactions. In summary, these results further demonstrate that the nanovaccine induced a systemic antitumor immune response to facilitate its immunotherapeutic effect, especially T cell-associated immune responses.

### The long-term immune memory effect generated by the nanovaccine inhibits tumor recurrence and metastasis

**Figure 7:**
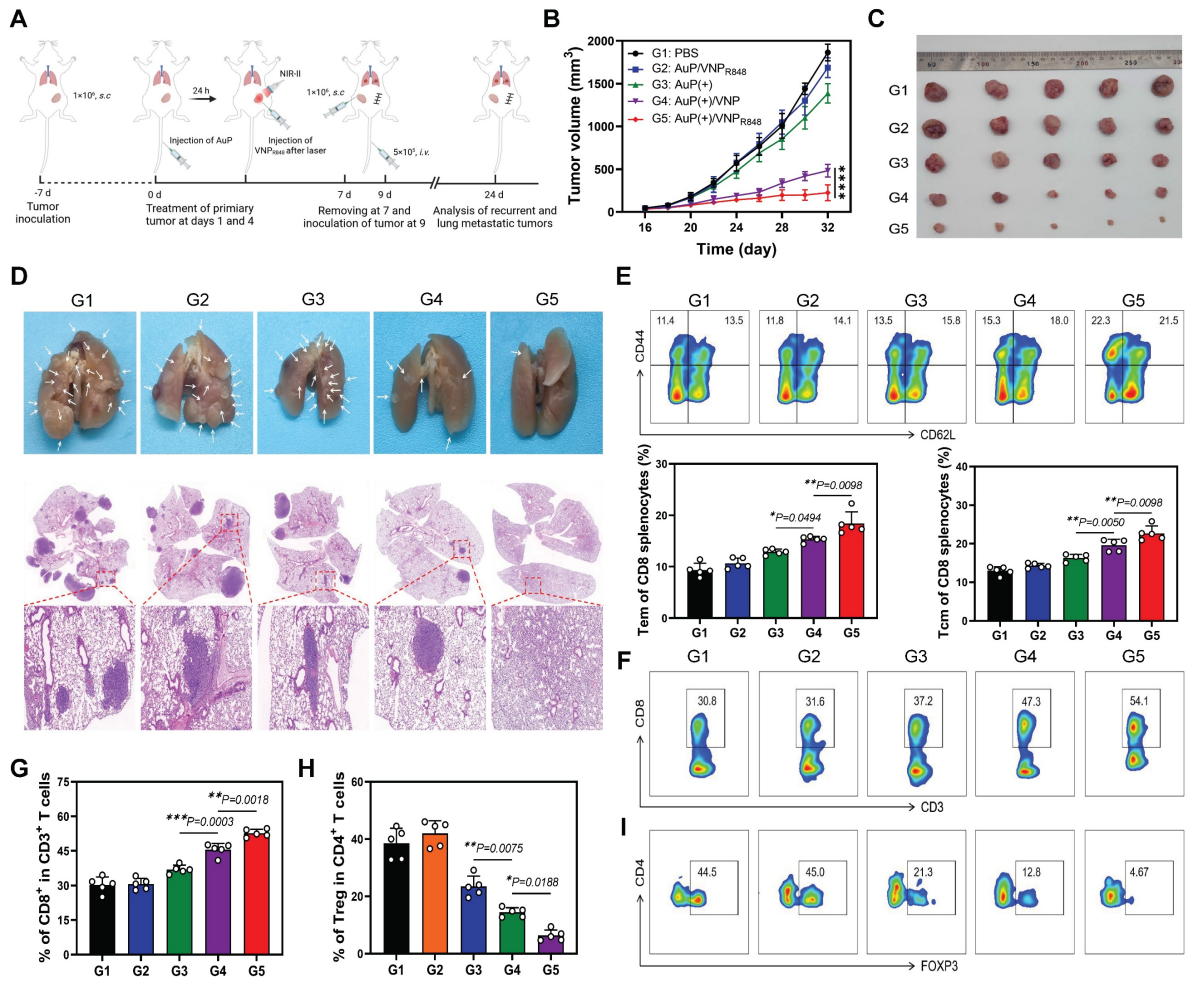
Long-term immune memory effects of tumor nanovaccines in inhibiting recurrent tumors and tumor metastasis. (A) Experimental protocol in recurrent and metastatic LLC tumor models. (B) LLC recurrent tumor growth curves in mice after various treatments (n = 5). (C) Photographs of *ex vivo* LLC tumors after various treatments. (D) Photographs and hematoxylin and eosin staining of lung metastatic nodules. (E) Representative flow cytometry images and relative quantification of effector memory T (Tem) cells and central memory T (Tcm) cells in the spleen (n = 5). (F, G) Representative flow cytometry analysis plots (F) and proportions (G) of CD8^+^ T cell (among CD45^+^CD3^+^ cells) (n = 5). (H, I) Quantitative statistical analysis (H) and representative flow cytometry plots (I) of Treg cells (FOXP3^+^CD4^+^ cells in CD45^+^CD3^+^ cells) (n = 5). Data are shown as the mean ± standard deviation. One-way ANOVA, *P < 0.05, **P < 0.01, ***P < 0.001, ****P < 0.0001. Image in A was created with BioRender.com.

The host immune system retains a memory of the presented antigen upon receiving immunotherapy. Consequently, immune system can recognize and respond more rapidly and robustly to subsequent antigen challenges, a phenomenon known as the immune memory effect 52-54. Inspired by the efficacy of the AuP(+)/VNP_R848_ nanovaccine in suppressing distant tumor growth, as demonstrated in the previous results, we proceeded to assess the efficacy of our AuP(+)/VNP_R848_-based nanovaccine in preventing tumor recurrence and lung metastasis. In our study, mice with primary subcutaneous LLC tumors were administered the AuP(+)/VNP_R848_ nanovaccine and other treatments on days 1 and 4. Subsequently, the tumors were surgically excised on day 7. To mimic tumor recurrence and lung metastasis, LLC cells were inoculated into the contralateral subcutaneous side and injected *i.v.* (Figure 7A). Compared to other groups, the AuP(+)/VNP_R848_ treatment group demonstrated the strongest suppression of recurrent tumors (Figure 7B and S20A), which was corroborated by tumor tissue images (Figure 7C) and tumor weight measurements (Figure S20B) obtained at the end of the experiment. Obviously, the AuP(+)/VNP_R848_ nanovaccine group exhibited the most effective suppression of lung metastasis compared with the other groups (Figure 7D, Figure S21 and S22). This was evidenced by a 95% remission in the amount of lung metastatic nodes in mice in the AuP(+)/VNP_R848_ nanovaccine group. Moreover, three mice showed no detectable metastatic foci in the AuP(+)/VNP_R848_ treatment group.

To delve deeper into the potential immune mechanisms of the nanovaccine in preventing tumor recurrence and metastasis and to ascertain if this inhibition was attributed to the immune memory effect initiated by the nanovaccine, the proportion of effector memory T (Tem) cells (CD44^+^CD62L^-^) and central memory T cells (Tcm) (CD44^+^CD62L^+^) within the spleen was assessed using multiparameter flow cytometry (Figure S23). The ratios of Tem and Tcm cells in the PBS group were the lowest, at 11.4% and 13.5%, respectively. The Au(+) and Au(+)/VNP groups exhibited moderate efficacy in generating Tem and Tcm cells, with Tem and Tcm of 13.5 and 15.8 for Au(+) and 15.3 and 15.8 for Au(+)/VNP, respectively. on the contrary, the ratios of Tem and Tcm cells in the Au(+)/VNP_R848_ group significantly raised to 22.3% and 21.5%, respectively (Figure 7E). Furthermore, the ratio of CD8^+^ T cells and Tregs within the recurrent tumors was also evaluated (Figure S24). Mice treated with AuP(+)/VNP_R848_ exhibited a peak CD8^+^ T cell proportion in recurrent tumors at 54.1%, the topmost among all the groups (Figure 7F-G). In contrast, the proportion of AuP(+)/VNP_R848_ immunosuppressive Treg cells was diminished to a minimum of 4.67% (Figure 7H-I). Notably, the survival analysis further confirmed the optimal treatment effect of AuP(+)/VNP_R848_. The results showed that 75% of the animals survived on day 60, whereas all mice in the other groups gradually succumbed within 55 days (Figure S25).

## Conclusion

To sum up, we developed a straightforward and innovative personalized cancer nanovaccine system called AuP/VNP_R848_. AuP, which has strong absorption in the NIR-II spectrum, generated a photothermal effect upon 1064 nm laser irradiation, effectively killing tumors *in situ* and converting tumor tissues into an antigen library by releasing abundant tumor antigens. Subsequently, VNP_R848_, owing to its ultrasmall size, efficiently captured tumor antigens, integrated with the immune adjuvant R848, and delivered tumor antigens to tumor-draining lymph nodes. VNP_R848_ is readily internalized by DCs, strongly stimulating their maturation and enhancing their ability to process antigens. Mature DCs then present the processed antigen-MHC complex to T cells, thus promoting T cell proliferation and activation. Simultaneously, R848 activates immune cells by triggering the TLR7/8 signaling pathway and upregulating antitumor cytokine secretion. Under the synergistic stimulation of these factors, the cancer nanovaccine AuP(+)/VNP_R848_ effectively initiated and amplified the immune response and achieved robust antitumor immune efficacy. This *in situ* cancer nanovaccine demonstrated excellent therapeutic effects against primary tumors, metastatic tumors, and recurrent tumor models while also inducing long-term immune memory *in vivo*. This approach provides a straightforward and effective method for designing novel personalized cancer nanovaccines for tumor immunotherapy.

## Experimental Section

*Preparation and characterization of AuP and VNP_R848_*: To synthesize AuP, Au nanorods (Au-NRs) were first synthesized. Freshly prepared NaBH_4_ (10 mM, 600 μL,) was added to a mixture of HAuCl_4_·3H_2_O (25 mM, 200 μL) and CTAB (0.1 M, 10 mL) as a seed liquid and left to stand for 90 min at 30°C to consume the excess NaBH_4_. Next, a growth liquid was prepared by blending CTAB (0.1 M, 20 mL) and HAuCl_4_·3H_2_O (25 mM, 400 μL) by stirring slowly, followed by adding AgNO_3_ (0.1 M, 120 μL) and mixing well. Subsequently, Hydroquinone (HQ) (0.1 M, 600 µL) was mixed to the growth liquid; when the color of the mixture disappeared (became colorless), 320 µL of the seed liquid was mixed to the growth liquid, and the reaction solution was stored under dark conditions for 12 h at 30°C. Excess HQ was removed via centrifugation at 500 ×*g* for 10 min. Finally, after centrifugation at 12000 rpm for 15 min at 30°C, the supernate was removed, and the sediment was resuspended in 15 mL of ultra-purified water to obtain Au-NRs. Subsequently, to prepare PEGylated gold nanorods (AuP), mPEG-SH (MW: 5000) and concentrated Au-NR (10 mM) solutions were gently blended, and the pH of the solution was regulated to 8.5 with a K_2_CO_3_ solution (0.1 M). After overnight incubation at 37°C, the sample was centrifuged four times with ultra-purified water to remove the unbound PEG.

VNP_R848_ was obtained empolying the single emulsification method. Specifically, 10 mg of mPEG-*b*-PHEP, 1 mg of DOTAP, and 1 mg of R848 were weighed and dissolved in 1 mL of chloroform solution. The solution was added to a 50-mL centrifuge tube containing 10 mL of ultra-purified water and ultrasonically emulsified applying a microtip probe sonicator (Bioruptor UCD 300, Diagenode) for 10 min (20% power), ‌subsequently reducing the pressure to remove chloroform and the unloaded R848 through a 220-nm filter membrane. The concentration of R848 was analysed using an ultraviolet spectrophotometer (UH5700, Shimadzu). The R848 encapsulation efficiency was calculated to be approximately 60%, with a loading capacity of 5%. The preparation process of NP_R848_ was similar to that of VNP_R848_, except that mPEG-PHEP was replaced with mPEG-PLA and the microtip probe power was set at 60%. Using a similar method, DiD-labeled VNP_DID_ was prepared by adding 4% (w/w) mPEG-*b*-PHEP to the formulation. The DiD concentration was analysed using a fluorescence spectrophotometer (F-7100, Hitachi). The size distribution, zeta potential, and stability in PBS of VNP_R848_ and NP_R848_ were investigated using a nanoparticle size analyzer (Malvern). The morphology of VNP_R848_ was observed via TEM (JEOLJEM-1400Plus).

*Young's modulus measurement:* 20 μL of NP_R848_ and VNP_R848_ solutions were dropped onto glass slides pre-coated with polyethylenimine (PEI) and left overnight to adhere to the slide surfaces. Using AFM (MultiMode 8-HR, Bruker) with a probe of spring constant 0.012 N/m, both nanoparticles were measured at a scanning rate of 0.1 Hz and a trigger force of 50 pN, and the Young's modulus of the two particles were calculated based on the measurement results.

*Cellular experiments*: To measure the uptake of VNP_R848_ by cells *in vitro*, BMDCs (5 × 10^5^ cells/well) were incubated with VNP_DID_ at different durations, and then the cells were harvested for flow cytometry analysis (BD FACSCelesta). For *in vitro* DC maturation, LLC cells (1 × 10^6^) were incubated using a Transwell system (seeded into the upper chamber) with various treatment formulations ([AuP] = 50 mg mL^-1^, [VNP_DID_] = 100 μg mL^-1^) in the presence or absence of 1064 nm laser irradiation (0.5 W cm^-2^, 10 min). BMDCs were added to the lower chamber and incubated for 24 h. After 24 h of co-culture, the BMDCs were harvested, stained on ice with PE-anti-mouse CD11c (Clone: N418), FITC-anti-mouse CD80 (clone: 16-10A1), and APC/Cy7-anti-mouse CD86 (Clone: GL-1) antibodies for 45 min, and analyzed via flow cytometer.

*Tumor antigen generation, capture, and discrimination in vitro*: LLC cells (2 × 10^5^ cells/well) and AuPs (50 mg mL^-1^) were incubated in a 24-well plate for 6 h, after which the medium was changed. Subsequently, LLC cells were treated with a 1064 nm laser (10 min, 0.5 W cm^-2^) and further incubated for 12 h. VNP_R848_ (100 μg mL^-1^) was then added to the cells and incubated for another 24 h. The total protein harvested for cell lysis was further filtered three times employing an ultrafiltration device via centrifugation at 1000 ×*g* for 3 min each time. Using DLS analysis to measure the particle size and zeta potential of VNP_R848_ after ultrafiltration. The capability of VNP_R848_ to capture antigen was tested by BCA protein quantification kit. VNPr848 captured protein was detected by sodium dodecyl sulfate polyacrylamide gel electrophoresis (SDS-PAGE) and Coomassie brilliant blue staining. To determine the quantity and type of protein, the protein was disintegrated into polypeptide fragments using trypsin, and the processed polypeptide sample was redissolved and measured by Liquid Chromatograph-Mass Spectrometer (LC/MS) to identify the type of VNP_R848_ captured protein. (LCMS-2010, Shimadzu).

*Lymph node accumulation*: LLC cells (1 × 10^6^) were injected subcutaneously into the backs of female C57BL/6 mice (6 weeks old) to establish a subcutaneous lung cancer model. When the tumor volume reached 100 mm^3^, the mice were randomly divided into two groups: PBS and VNP_DID_. VNP_DID_ was injected *i.t.* at a dose of 2.5 μg per mouse. After 24 h of administration, the tumor-draining lymph nodes were collected and the DiD fluorescence signal was tested using an IVIS spectroscopy system (IVIS Lumina III, PerkinElmer). Subsequently, the harvested lymph nodes were ground, disaggregate into single-cell suspensions, stained with BV510-anti-mouse CD45 (Clone: M1/70) and PE-anti-mouse CD11c (Clone: N418) antibodies, and investigated using flow cytometry.

*Antitumor efficacy analysis*: Mice bearing subcutaneous LLC tumors (tumor volumes ~100 mm^3^) were randomly divided into six groups: (G1) PBS, (G2) AuP, (G3) AuP/VNP_R848_, (G4) AuP(+), (G5) AuP(+)/VNP, and (G6) AuP(+)/VNP_R848_. AuP was injected *i.v.* before 24 h of laser irradiation, while the primary tumors treated with VNP_R848_ *i.t.* injection. Mice treated with AuP(+) were then irradiated with a 1064 nm laser at 24 h post-administration (0.5 W cm^-2^, 10 min). Temperature changes in the tumors were recorded using an infrared camera. To detect DC maturity and cytokine production *in vivo*, the mice were euthanized after receiving various treatments. Their tumor-draining lymph nodes were then isolated and processed via mechanical grinding, passed through a 200-mesh nylon mesh to create single-cell suspensions, and incubated with BV510-anti-mouse CD45 (clone: 104), PE-anti-mouse CD11c (clone: N418), FITC-anti-mouse CD80 (clone: 16-10A1) and APC/Cy7-anti-mouse CD86 (clone: GL-1) antibodies for flow cytometric analysis. Simultaneously, the collected mouse peripheral serum was detected using ELISA kits (IL-6, IL-12p70, TNF-α, IFN-γ) on the basis of standard protocols.

To establish a distal lung cancer tumor model, LLC cells (1 × 10^6^) were injected subcutaneously on both sides of the backs of female C57BL/6 mice, and the mice (with a tumor volume of ~100 mm^3^) were randomly divided into the same six groups as described above (n=5 mice in each group) after 7 days. The primary tumor of mice treated with AuP(+) was irradiated with a 1064 nm laser at 24 h post-administration on days 1 and day 4 (10 min, 0.5 W cm^-2^), and subsequently injected with VNP_R848_ *i.t*. Bilateral tumor volume was recorded by measuring the perpendicular diameter using a Vernier caliper. The tumor volume and mouse body weight were monitored every two days, and the resulting serum was tested using an automatic biochemical analyzer (Hitachi 3100). To establish a mouse model of tumor recurrence and lung metastasis, 1 × 10^6^ LLC cells were injected subcutaneously into the right side of the backs of female C57BL/6 mice. After seven days, when the primary tumor volume reached 100 mm^3^, the mice were randomly divided into five groups (n=5 mice per group). AuP/VNP_R848_, AuP(+), AuP(+)/VNP, and AuP(+)/VNP_R848_ were administered to four groups of mice on days 1 and 4. Primary LLC tumors were surgically excised on day 7, and LLC cells were inoculated on the other subcutaneous side and injected into the tail vein on day 9 to simulate tumor recurrence and lung metastasis. On day 24, the lungs of the sacrificed mice were obtained, photographed, and fixed in 4% paraformaldehyde for H&E staining.

*Intratumoral lymphocyte and spleen immune memory effect analysis*: Distant tumor tissue was obtained from the distal lung cancer tumor model, minced, and digested with RPMI-1640 medium containing 10% fetal bovine serum, type IV collagenase (1 mg mL^-1^), hyaluronidase (100 μg mL^-1^), and DNase I (100 μg mL^-1^) at 37°C and 150 rpm for 20 min. The assimilated cells were then filtered through a nylon mesh (200-mesh), harvested via centrifugation (600 ×*g*, 5 min), and further purified using a 40% Percoll (GE) solution. The acquired lymphocytes were collected and incubated with BV510-anti-mouse CD45 (clone: 104), FITC-anti-mouse CD3 (clone: 17A2), APC/Cy7-anti-mouse CD4 (clone: GK1.5), PE-anti-mouse CD8a (clone: S18018E), BV421-anti-mouse Foxp3 (Clone: MF-14), APC-anti-mouse IFN-γ (clone: XMG1.2), and AF700-anti-mouse KI67 (clone: 16A8) for flow cytometric analysis.

To explore the immune memory effect of the *in situ* nanovaccine, single-cell suspensions of spleen tissue from the LLC pulmonary metastasis model were collected and incubated with BV510-anti-mouse CD45 (clone number: 104), APC/Cy7-anti-mouse CD4 (clone: GK1.5), PE-anti-mouse CD8a (clone: S18018E), BV421-anti-mouse CD3 (clone: 17A2), APC-anti-mouse CD44 (clone: IM7) and FITC-anti-mouse CD62L (clone: MEL-14) antibodies to investigate the proportion of Tem and Tcm cells by flow cytometric analysis.

*Transcriptomic analysis*: Distal tumors were harvested from the distal lung cancer tumor models. Total RNA was abstracted according to manufacturer's illustration. RNA-sequencing analysis was then performed after measuring the RNA concentration and integrity using an Agilent 2100 Bioanalyzer system (Agilent Technologies). Differentially expressed genes were identified using the DESeq R package (4.0.0) with the following threshold conditions: p-value < 0.05, and |log2(FoldChange)| > 0. Volcano and heat maps were generated using the ggplot2 and pheatmap packages in R, respectively. GO and KEGG enrichment analyses were performed using ClusterProfiler (3.8.1), and bubble plots were drawn using the ggplot2 package in R. Significant enrichment was identified using a p-value of < 0.05. Protein-protein interaction networks were generated using Cytoscape software.

## Supplementary Material

Supplementary methods and figures.
