# Supplementary material for: NIR-II light-driven in situ nanovaccine for cancer immunotherapy via lymph node migration-mediated accumulation
Source: Theranostics. 2025 Jul 2;15(15):7677–92. doi: 10.7150/thno.114347 (PMC12316039; doi:10.7150/thno.114347)
Supplement: Supplementary file 1 — Supplementary methods and figures. [file thnov15p7677s1.pdf]

Supplementary methods and figures.

NIR-II light-driven in situ nanovaccine for cancer immunotherapy via lymph node migration-mediated accumulation

Yucheng Huang[1,2#], Miao Su[3#], Weihuan Lin[4#], Peirong Zhang[2,5], Hengliang Hou[1,2], Fanjun Zeng[4], Luyu Huang[6], Qiaxuan Li[2], Jialong Deng[1], Shengbo Liu[1], Hongrui Qiu[2], Xiaoqing Yuan[7], Li Peng[7], Bin Xu[3]*, Haiyu Zhou[1,2]**

1. School of Medicine, South China University of Technology, Guangzhou, 510006, P. R. China

2. Department of Thoracic Surgery, Guangdong Provincial People's Hospital (Guangdong Academy of Medical Sciences), Southern Medical University, Guangzhou, 510080, P. R. China

3. School of Biomedical Sciences and Engineering, South China University of Technology, Guangzhou International Campus, Guangzhou, 511442, P. R. China

4. Department of General Practice, Guangdong Provincial Geriatrics Institute, Guangdong Provincial People's Hospital (Guangdong Academy of Medical Sciences), Southern Medical University, Guangzhou, 510080, P. R. China

5. Department of Thoracic Surgery, Maoming People's Hospital, Maoming, 525000, P. R. China

6. Department of Surgery, Competence Center of Thoracic Surgery, Charité Universitätsmedizin Berlin, Berlin, 10117, Germany

7. Medical Research Center, Sun Yat-Sen Memorial Hospital, Sun Yat-Sen University, Guangzhou, 510120, P. R. China.

[#] These authors contributed equally to this work.

** Address correspondence to: zhouhaiyu@gdph.org.cn (H. Zhou), bsbun_hui@mail.scut.edu.cn (B. Xu), pengli9@mail.sysu.edu.cn (L. Peng)*

Supplementary Methods

Materials. Silver nitrate ($AgNO_3$), sodium borohydride ($NaBH_4$), hydroquinone, Poly(ethylene glycol) methyl ether thiol (mPEG-SH, MW: 5000), hyaluronidase, DNase I, DiD were purchased from Sigma-Aldrich (Missouri, USA). Cetyltrimethylammonium bromide (CTAB) and tetrachloroauric (III) acid trihydrate ($HAuCl_4 \cdot 3H_2O$) were purchased from Aladdin (Shanghai, China). R848 was purchased from MedChemexpress Biotechnology (New Jersey, USA). Dulbecco's modified eagle's medium (DMEM), trypsin-EDTA (0.25%) and Roswell park memorial institute-1640 (RPMI-1640) were purchased from Gibco (Grand Island, USA). Penicillin-streptomycin solution (100×) were purchased from Corning Life Sciences Co., Ltd. (Suzhou, China). Fetal bovine serum (FBS) was purchased from ExCell Bio (Shanghai, China). Hematoxylin and eosin (H&E) staining kit and proteinase K were purchased from Beyotime (Shanghai, China). 10 × red blood cell lysis solution and all flow cytometry antibodies were purchased from BioLegend (San Diego, USA).

Cell lines and animals. The murine lung cancer cell line LLC and DC 2.4 cell lines were obtained from the American Type Culture Collection (ATCC). LLC cells were cultured in DMEM medium, and DC 2.4 cells were cultured in RPMI-1640 medium, both of which were supplemented with 10% FBS and 1% penicillin-streptomycin at 37 °C using a humidified 5% CO_2 incubator. C57BL/6 female mice (6 weeks) were purchased from Guangdong Zhiyuan Biopharmaceutical Technology Co., Ltd. (Guangdong, China). All animal studies in accordance with the animal protocol were approved by South China University of Technology Animal Care and Use Committee and the Guangdong Provincial People's Hospital (Guangdong Academy of Medical Sciences) (Approved number: KY2024-347-01).

Photothermal effect of AuP. The aqueous solution of AuP at different concentrations

(25, 50 and 100 μg mL^{-1}) were irradiated with a 1064 nm laser with a laser power of 0.5 W cm^{-2}. A near-infrared camera was used to record the temperature changes for 10 min. Moreover, AuP (50 μg mL^{-1}) were irradiated with 0.5 W cm^{-2} or 1.0 W cm^{-2} lasers to monitor temperature change. In addition, laser irradiation was repeated three times to indicate the photothermal stability of AuP.

***In vitro* cytotoxicity of AuP and VNP$_{R848}$.** LLC cells were incubated with different concentrations of AuP for 4 h and then irradiated with a 1064nm laser (0.5 W cm^{-2}, 10 min). After a further 4 h of incubation, cell viability was measured using Cell Counting Kit-8. DC2.4 or BMDC cells were seeded into 96-well plates and incubated with different concentrations of VNP$_{R848}$. After 24 h of incubation, cell viability was assessed using the Cell Counting Kit-8 assay.

Culture of bone marrow-derived dendritic cells (BMDCs). BMDCs were generated from the C57BL/6 mice. The bone marrow cells were flushed out of the femurs and tibias with sterile PBS and filtered with a cell strainer (200 mesh) to obtain single cell suspensions. After being purified through red blood cell lysis, the cells were cultured with RPMI 1640 containing GM-CSF (20 ng mL^{-1}) and IL-4 (10 ng mL^{-1}) at 37 °C, and the medium was replaced with an equal volume on day 3. On day 6, BMDCs were collected for further experiments.

Biodistribution and accumulation *in vivo*. LLC tumor-bearing mice were injected with AuP via the tail vein, and free R848 or VNP$_{R848}$ were injected intratumorally ([AuP]= 1 mg kg^{-1}, [VNP$_{R848}$]= 30 mg kg^{-1}) . The organs, tumors and lymph nodes were harvested and grind after 24 hours post-administration, followed Au and R848 were measured by ICP-OES (iCAP 7200 Duo, Thermofisher Scientific, USA) and UPLC (H-Class, Waters, USA), respectively.

***In vivo* Photoacoustic microscop imaging of AuP.** For *in vivo* PAM imaging, LLC tumor-bearing mice were treated with AuP (1 mg kg^{-1}) via tail vein injection. PAM images were acquired at designated time points (0, 4, 12, 24, and 36 hours post-injection) using a PA imaging system (PASONO-ANI, Guangdong Photoacoustic Technology). The PAM images were obtained under laser irradiation with 532 nm excitation wavelength (18 mJ cm^{-2}) and 1064 nm excitation wavelength (80 mJ cm^{-2}). The resolution of the rastor scanning was 256 x 256 pixels, and three-dimensional volumetric PAM data were acquired via optical scanning. MATLAB (R2014b, MathWorks) was used to generate three-dimensional volumetric visualization. Full-field photoacoustic images (10 × 10 mm^2) were selected to monitor tumor signal variations.

Statistical Analysis. All results are expressed as mean ± s.d.. Statistical analysis was analyzed using an unpaired Student's t-test (two-tailed) for two groups, and one-way ANOVA with Tukey's multiple comparisons. Significance levels were defined as n.s.: no significant difference, $P > 0.05$, $^*P < 0.05$, $^{**}P < 0.01$, $^{***}P < 0.001$, and $^{****}P < 0.0001$, P values were calculated by GraphPad Prism v9.0.0 (GraphPad Software, San Diego, CA) and marked on the figures.

Supplementary Figures

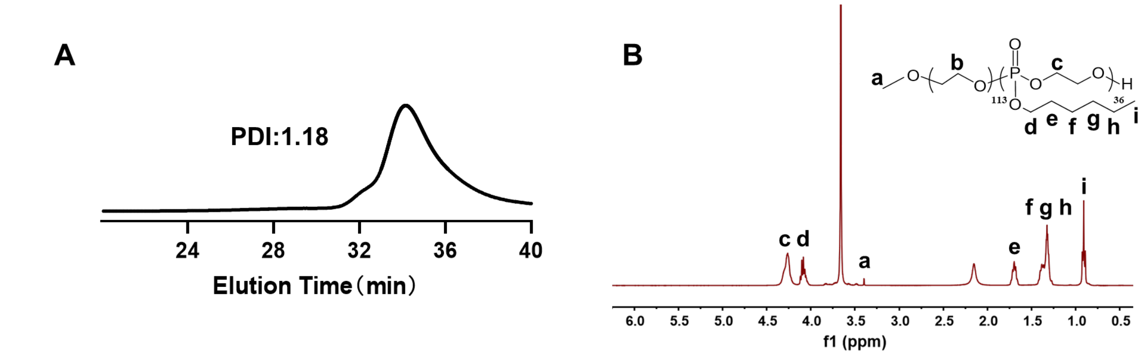

Figure S1. Synthesis and characterization of the amphiphilic poly-(ethylene glycol)-*block*-poly(2-hexoxy-2-oxo-1,3,2-dioxaphospholane (mPEG-*b*-PHEP). (A) GPC spectrum of mPEG-*b*-PHEP was determined on a gel permeation chromatography (GPC) system (Waters, USA) with a DMF mobile phase at a speed of 0.3 mL min-1. Polydispersion index (PDI): 1.18. (B) ^{1}H NMR spectrum of mPEG-*b*-PHEP was determined by AVANCE III 600 MHz NMR spectrometer (Bruker,Switzerland) in $CDCl_3$.

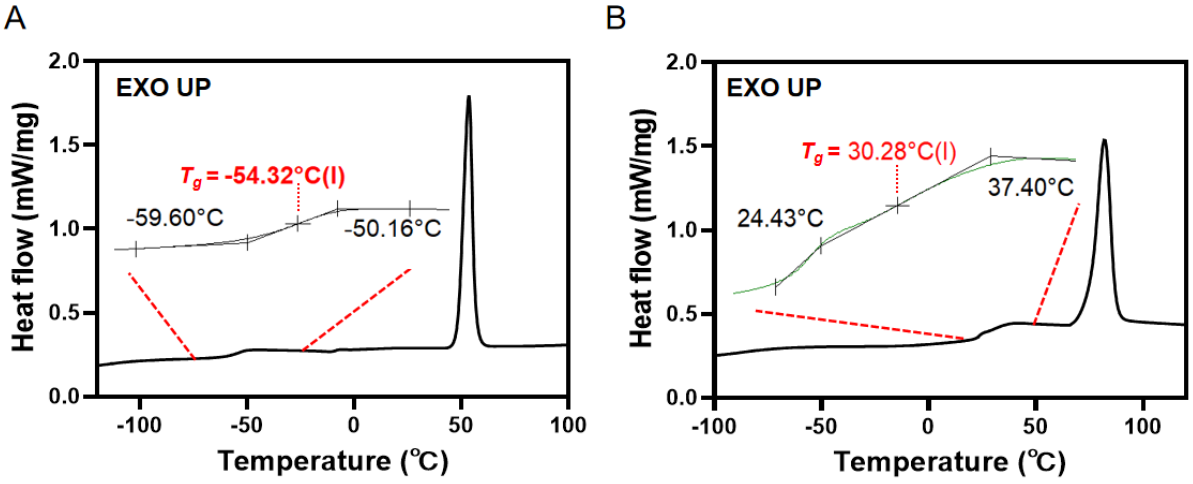

Figure S2. Differential scanning calorimetry (DSC) heating curves of mPEG-PHEP (A) and mPEG-PLA (B). The glass transition temperatures (T_g) were measured using a DSC200 F3 (NETZSCH) under N_2 at a heating rate of 10 °C/min. The T_g corresponds to the midpoint of the inflection point tangent in the curve.

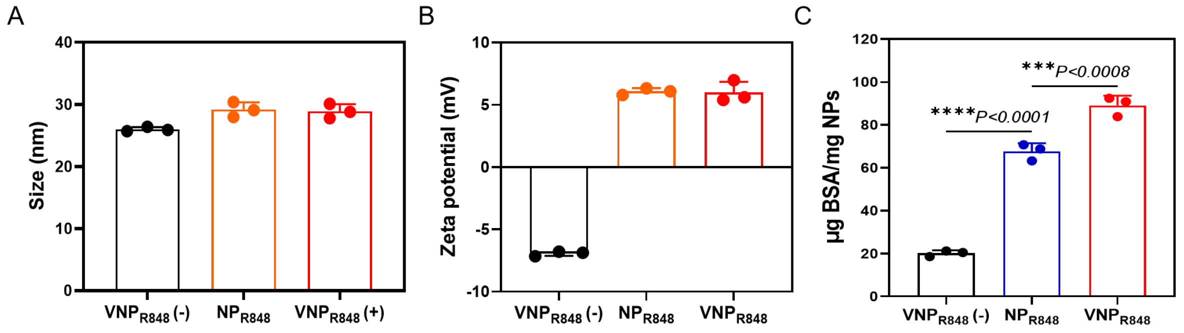

Figure S3. The number sizes (A) and zeta potentials (B) of NP_{R848} and VNP_{R848} nanoparticles with or without the cationic liposome DOTAP. (C) The protein capture capabilities of NP_{R848} and VNP_{R848} with or without the cationic liposome DOTAP. Data are presented as mean s.d. Statistical significance was analyzed by one-way ANOVA with Tukey's multiple comparisons test. ***$P < 0.001$; ****$P < 0.0001$.

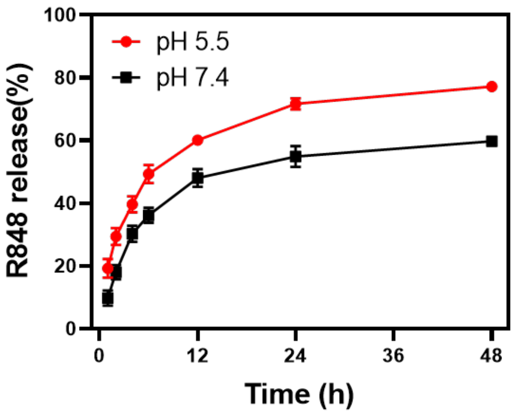

Figure S4. The R848 release kinetics of VNP_{R848} in PBS at pH 5.5 and 7.4.

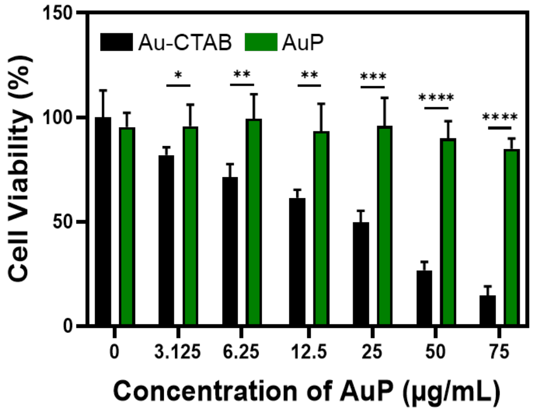

Figure S5. Cytotoxicity of gold nanorods before and after PEGylation. Data are presented as mean s.d.; the comparison of two groups was followed by Unpaired student's t-test (two-tailed). *P < 0.05; **P < 0.01; ***P < 0.001; ****P < 0.0001.

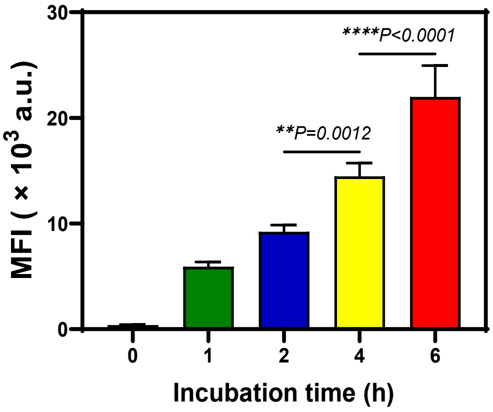

Figure S6: BMDC uptake of VNP_{R848} at different times. Data are presented as mean s.d. Statistical significance was analyzed by one-way ANOVA with Tukey's multiple comparisons test. **P < 0.01; ****P < 0.0001.

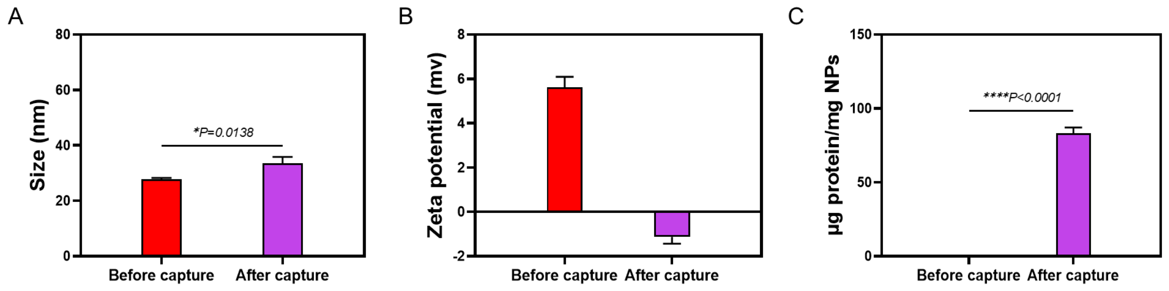

Figure S7. Characteristic of antigen capture of VNP_{R848}. (A, B) Size (A) and zeta potential (B) of VNP_{R848} after capturing proteins released from cells. (C)The protein content adsorbed by VNP was measured by BCA Protein Quantification kit (n = 3). Data are presented as mean s.d.; the comparison of two groups was followed by Unpaired student's t-test (two-tailed). *P < 0.05; ****P < 0.0001.

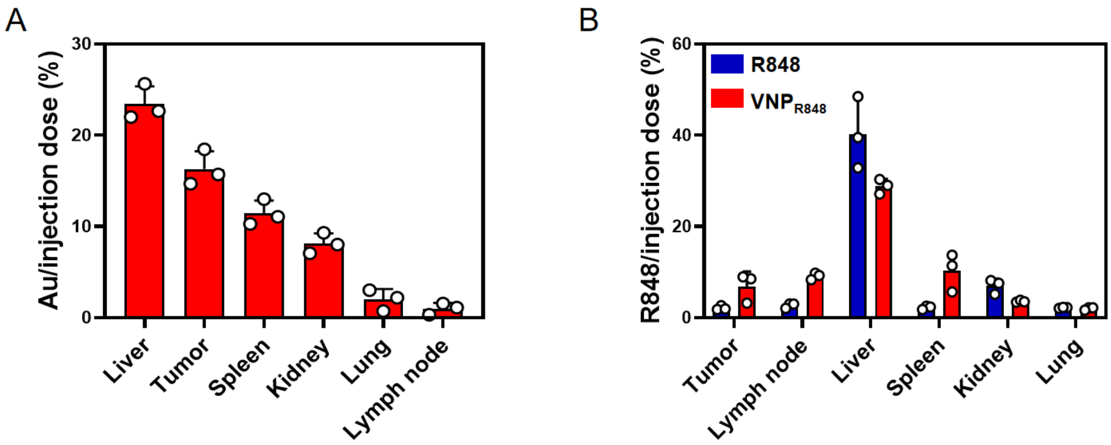

Figure S8. Quantification of Au, R848 in lymph node and main organs. Quantitative analyses Au (A) and R848, VNP_{R848} (B) distribution in main organs.

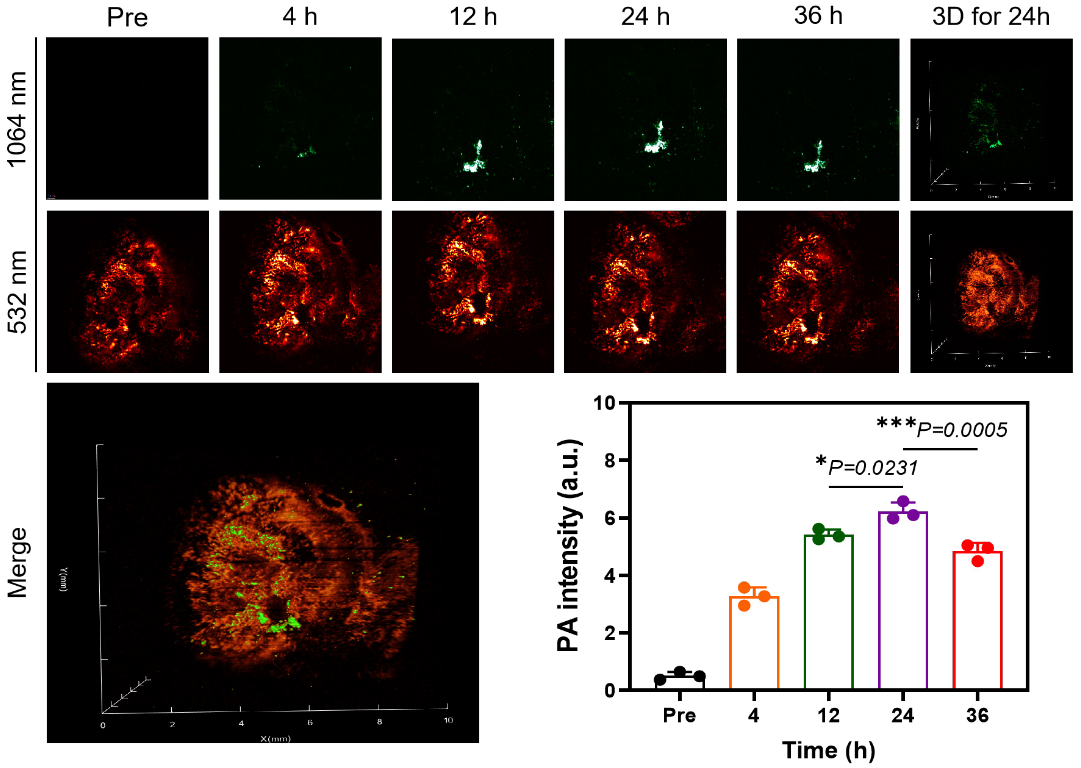

Figure S9. *In vivo* PAM imaging of AuP. *In vivo* PAM images and average PA intensity of LLC tumors before and at different monitoring time points after administration of AuP. Data are presented as mean s.d. Statistical significance was analyzed by one-way ANOVA with Tukey's multiple comparisons test. *P < 0.05; ***P < 0.001.

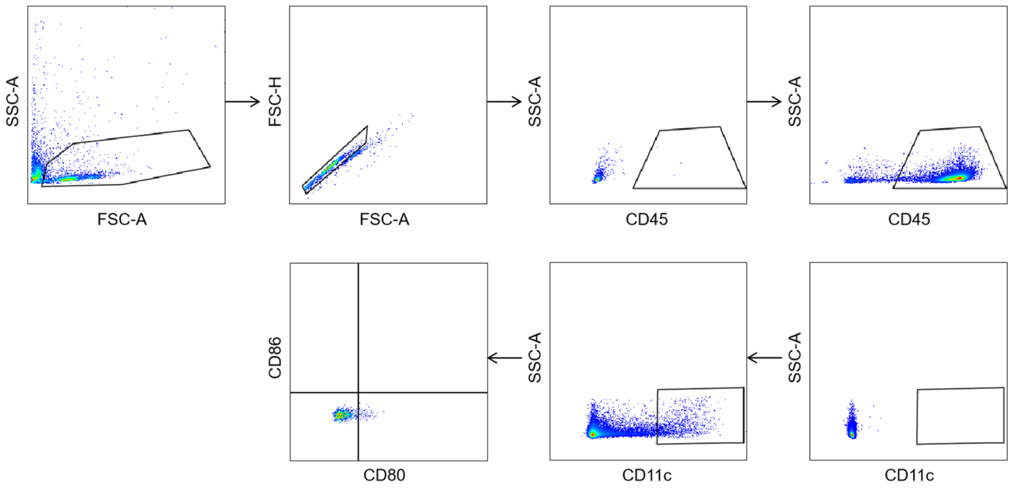

Figure S10. Gating Strategy to identify $CD80^{+}CD86^{+}$ DC cells maturity percentage. Cell populations were gated sequentially following arrows.

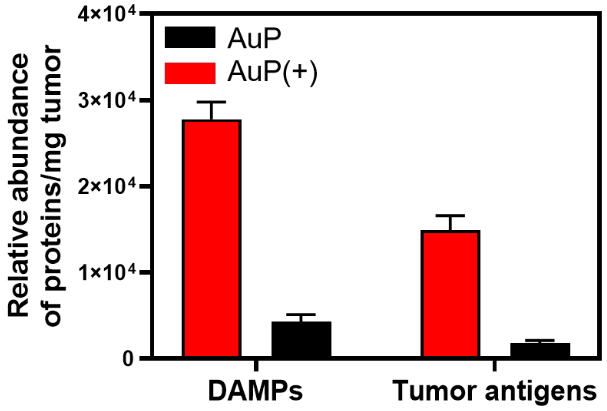

Figure S11. The relative abundance of DAMPs and tumor antigens generated per milligram of tumor tissue. Data are presented as mean s.d.

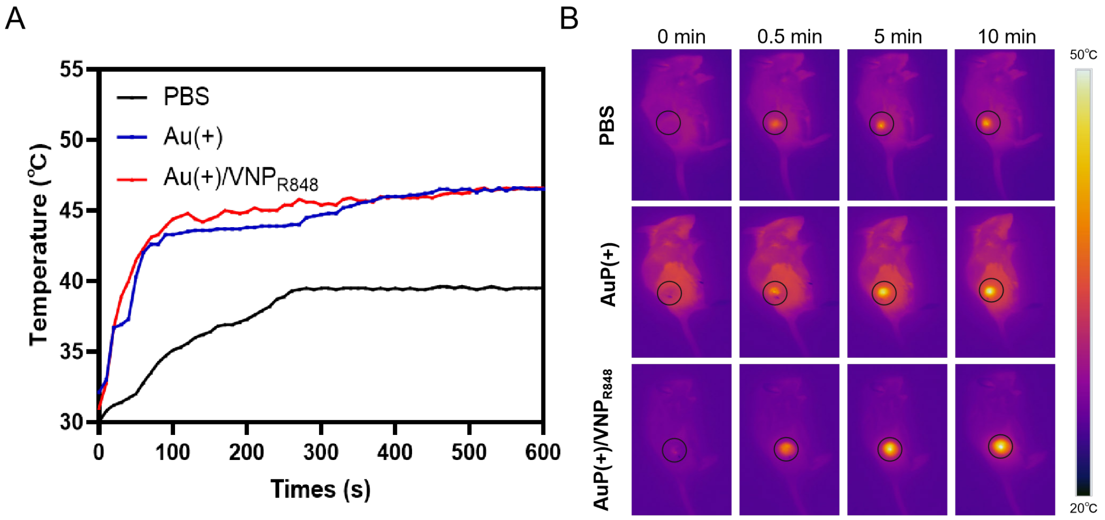

Figure S12. *In vivo* photothermal effect of AuP/VNP$_{R848}$ under 1064 nm illumination. (A, B) Photothermal curve (A) and images (B) of AuP and AuP/VNP$_{R848}$ under 1064 nm illumination (0.5 W cm^{-2}, 10 min) *in vivo*. The Photothermal images were recorded by infrared camera (FOTRIC, China) and analyzed by FOTRIC AnalyzIR software v4.4.

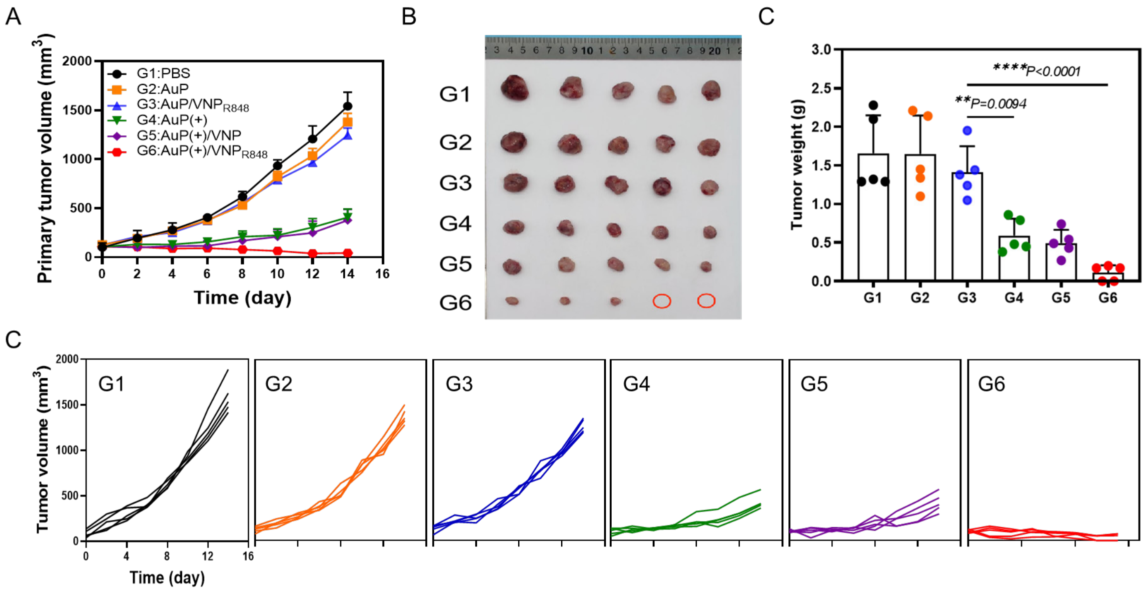

Figure S13. Antitumor effect of nanovaccines in primary tumor (A) Average primary tumor growth curves of LLC tumor-beating mice after various treatments (n = 5). (B) Images of primary tumor after various treatments. (C) Primary tumor weight of various groups at the end point of the treatments. (D) Individual primary tumor growth curves of LLC tumor beating mice after various treatments (n = 8). Data are presented as mean s.d. Statistical significance was analyzed by one-way ANOVA with Tukey's multiple comparisons test. **P < 0.01; ****P < 0.0001.

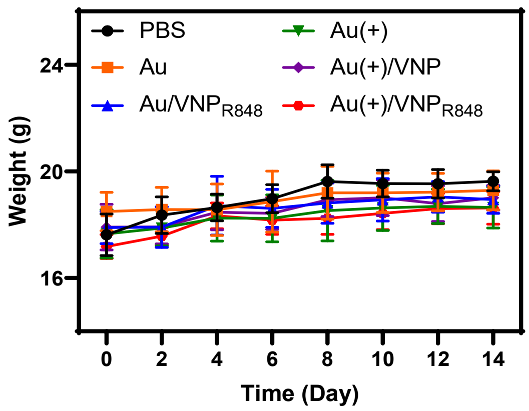

Figure S14. Body weight of mice bearing LLC tumor during receiving various treatment.

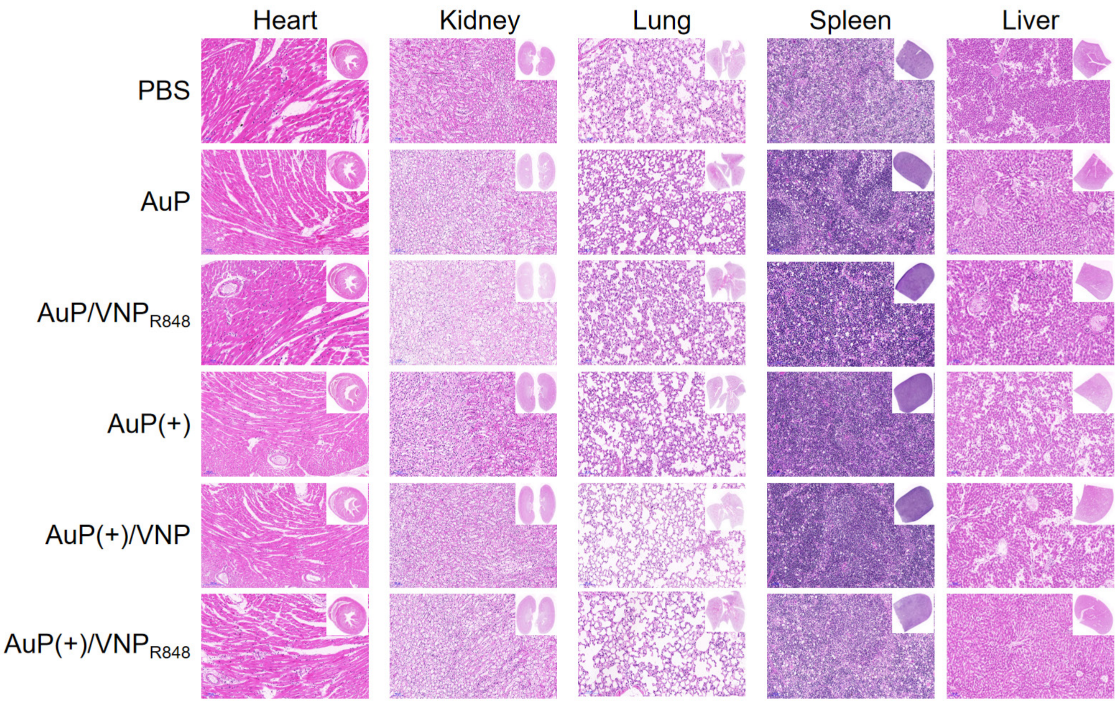

Figure S15. *In vivo* safety assessed by histopathological analysis. Representative hematoxylin and eosin (H&E) staining of tissue sections from major organs of mice after treatment with various treatment.

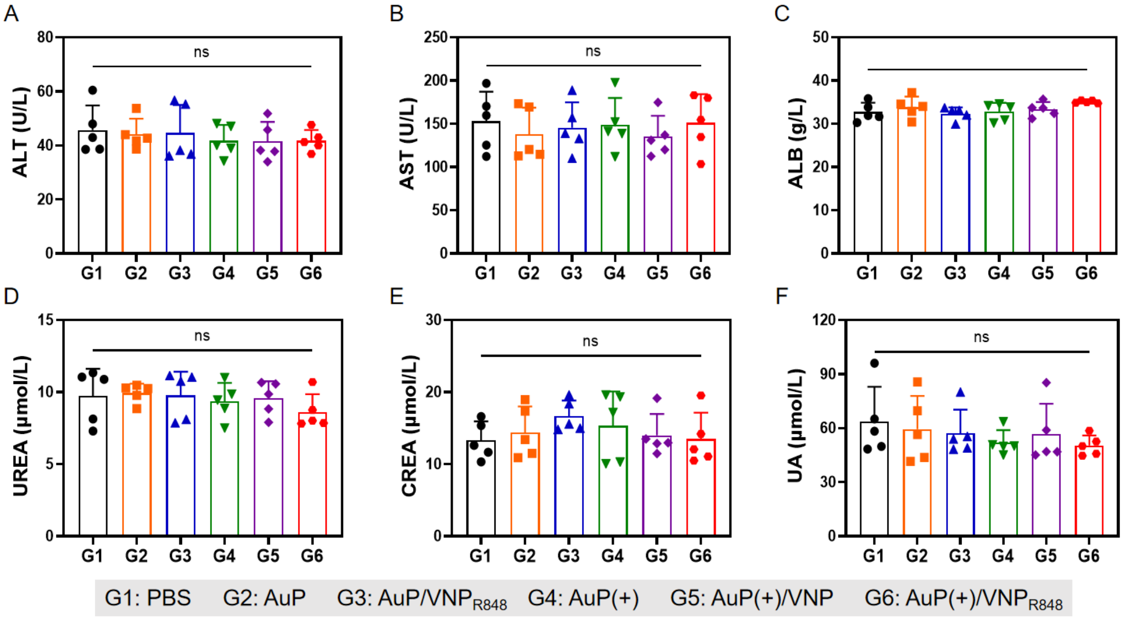

Figure S16. Blood biochemistry test of mice bearing LLC tumor after receiving various

treatment (n = 5). (A) ALT: alanine aminotransferase. (B) AST: aspartate aminotransferase. (C) ALB: albumin. (D) UREA: ureatinine. (E) CREA: creatinine. (F) UA: uric acid. Data are presented as mean s.d..

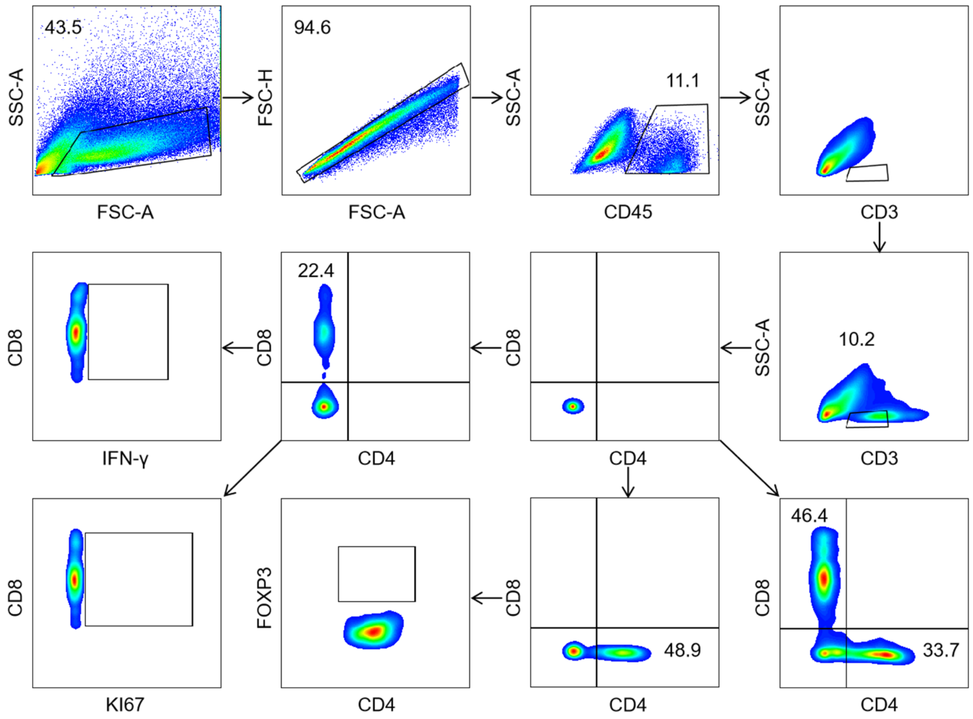

Figure S17. Gating Strategy to identify $CD8^+$ T cells percentage, $ki67^+CD8^+$ T cells percentage, INF-γ^+CD8^+ T cells percentage and Treg cells percentage. Cell populations were gated sequentially following arrows.

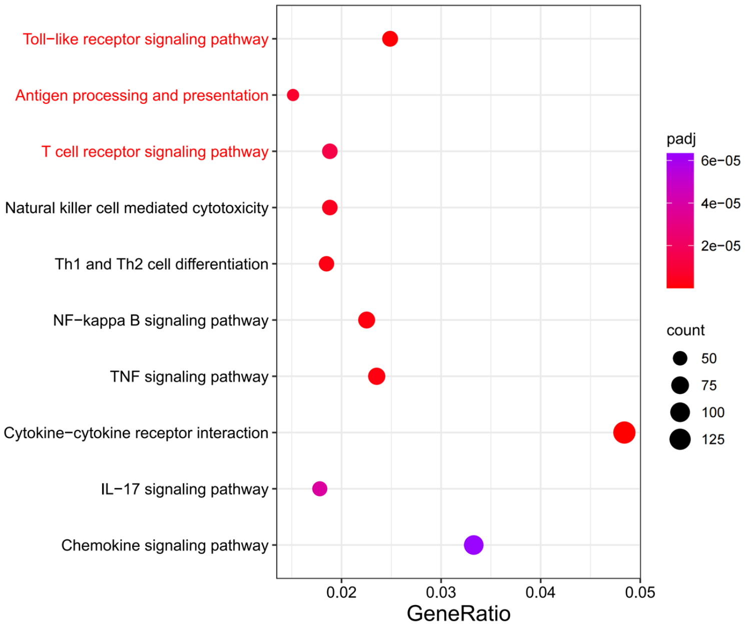

Figure S18. KEGG pathway analysis of the significantly upregulated genes in the AuP(+)/VNP$_{R848}$ group compared with the PBS group.

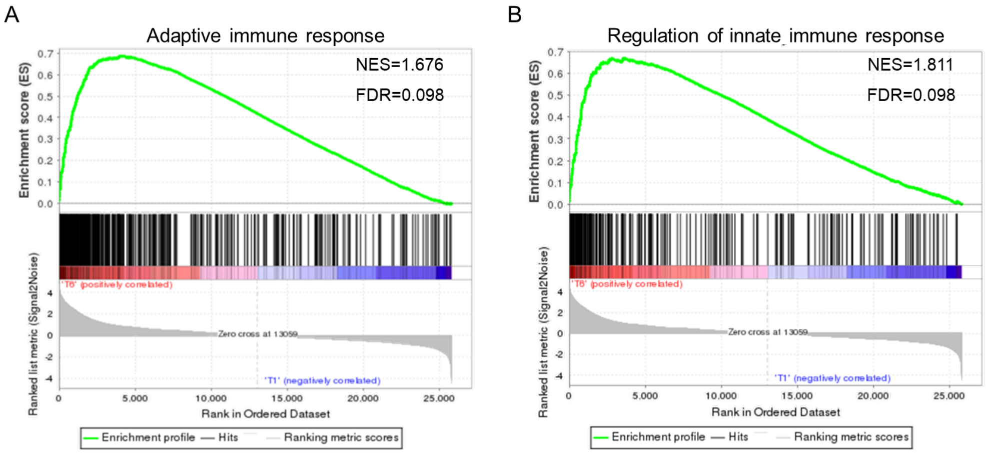

Figure S19. Gene set enrichment analysis (GSEA) for (A) adaptive immune response and (B) Regulation of innate immune response after AuP(+)/VNP$_{R848}$ treatment.

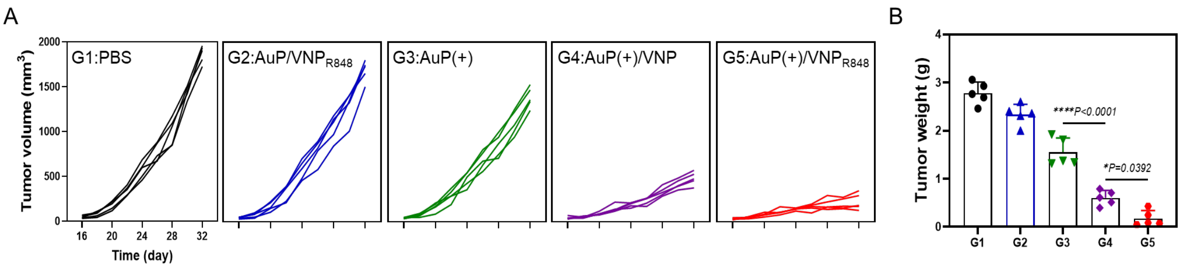

Figure S20. (A) Individual tumor growth curves of LLC tumor-bearing mice after various treatments (n = 5). (B) Tumor weight of various groups at the end point of the treatments. Data are presented as mean s.d.Statistical significance was analyzed by one-way ANOVA with Tukey's multiple comparisons test. *P < 0.05; ****P < 0.0001.

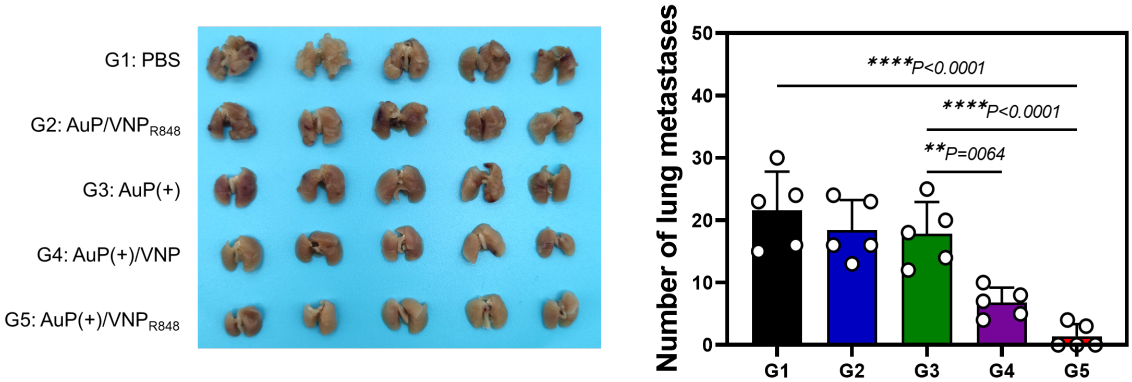

Figure S21. Photographs of lung metastatic nodules and statistics on the number of lung nodules at the end of treatment. Data are presented as mean s.d.Statistical significance was analyzed by one-way ANOVA with Tukey's multiple comparisons test. **P < 0.01; ***P < 0.001; ****P < 0.0001.

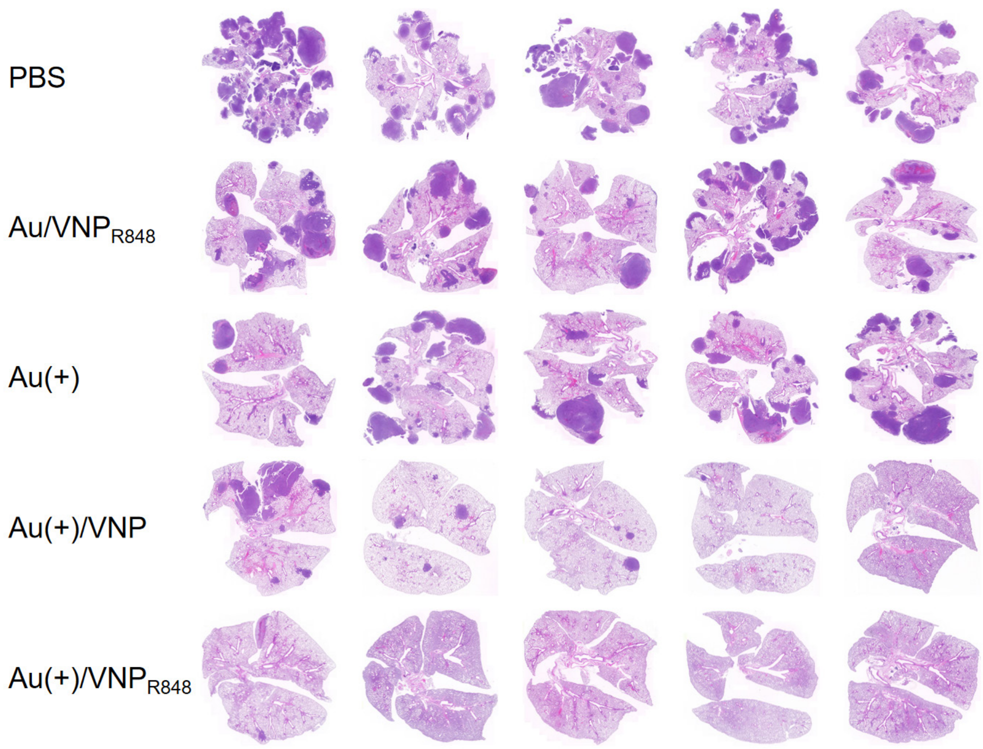

Figure S22. H&E staining of lung metastatic nodules.

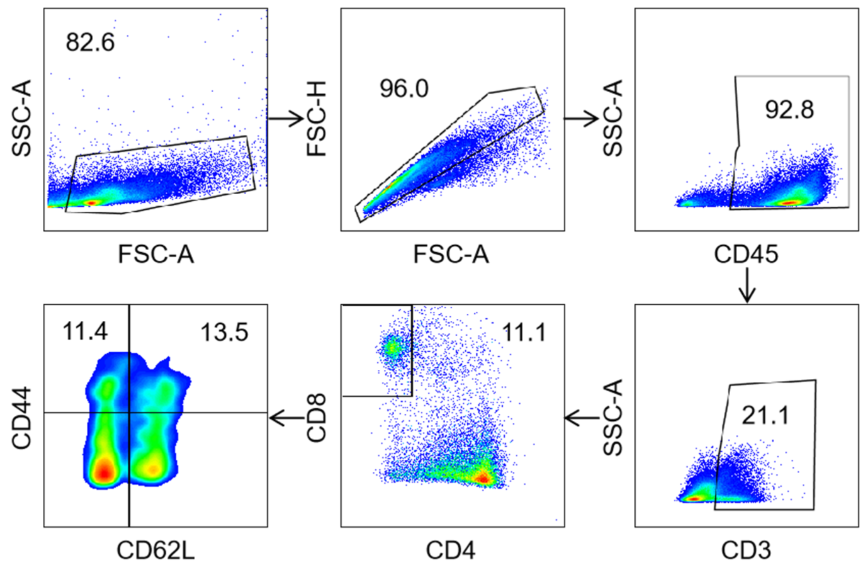

Figure S23. Gating strategy to identify effector memory T cells (Tem) and central memory T (Tcm) cells in spleen. Cell populations were gated sequentially following arrows. Tem cells were gated as $CD45^+CD3^+CD8^+CD44^+CD62L^-$ and Tcm cells were gated as $CD45^+CD3^+CD8^+CD44^+CD62L^+$.

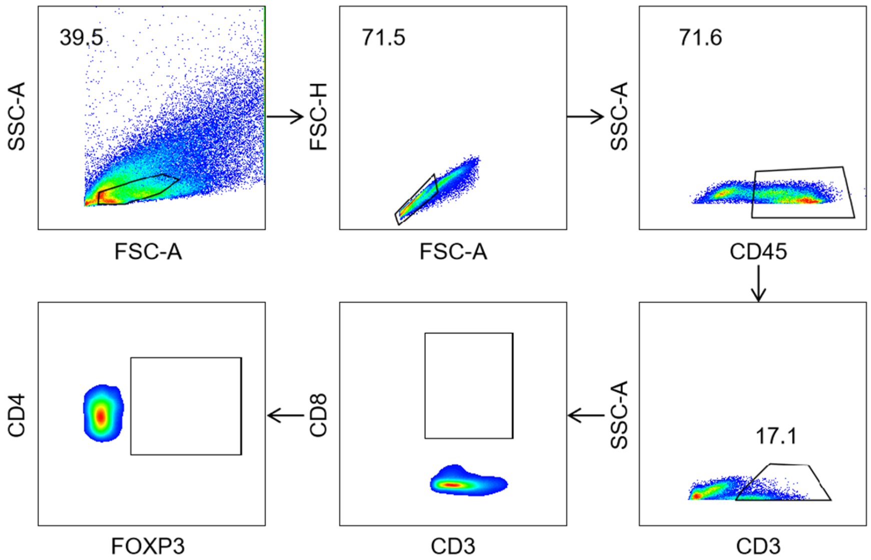

Figure S24. Gating Strategy to identify CD8^{+} T cells percentage and Treg cells percentage. Cell populations were gated sequentially following arrows.

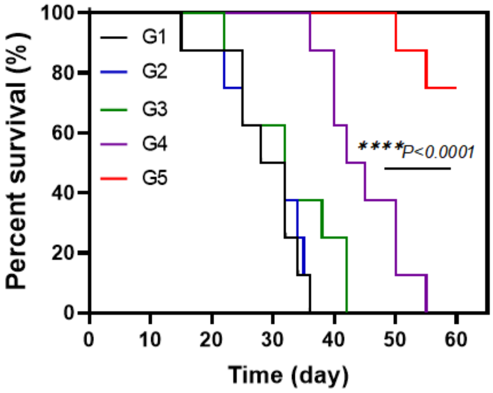

Figure S25. Survival rates of mice treated in different groups (n = 8). The statistical significance of the survival was calculated via log-rank (Mantel-Cox) test. ****P<0.0001.
